# Differential adaptations of japonica rice to submergence stress during the tillering stage under various seedling cultivation and transplanting methods

**DOI:** 10.3389/fpls.2025.1607055

**Published:** 2025-06-19

**Authors:** Sumei Duan, Qianxi Zhang, Hao Ai, Tingting Feng, Aifeng Zhou, Yi Liu, Yuqin Wang, Fei Fang

**Affiliations:** ^1^ College of Agriculture, Anhui Science and Technology University, Chuzhou, China; ^2^ Anhui Xin Fu Xiang Tian Ecological Agriculture Co. Ltd., Ma’anshan, China; ^3^ Ma’anshan Agriculture and Rural Bureau, Ma’anshan, China

**Keywords:** tillering, cultivation, tolerance, rice, submergence

## Abstract

The response mechanisms of rice to submergence stress during the tillering stage remain unclear, and different seedling cultivation and transplanting methods may influence submergence tolerance. This study aimed to investigate the differential responses of rice to submergence stress under diverse combinations of seedling cultivation and transplanting methods, providing a theoretical basis for evaluating rice submergence tolerance. The japonica rice cultivar 'Nanjing 46' was used as the test material. Five combinations of seedling cultivation and transplanting methods (Y1–Y5, including direct seeding and hard-ground dry nursery substrate micro-sprinkler tray seedling combined with machine transplanting) were established, combined with four submergence durations (0 days/B0, 4 days/B1, 7 days/B2, and 10 days/B3), resulting in 20 treatments. Agronomic traits (plant height, tiller survival rate), physiological indices [peroxidase (POD) and superoxide dismutase (SOD) activities, malondialdehyde (MDA) and proline (PRO) contents, soil and plant analyzer development (SPAD) value], and yield data were analyzed to evaluate submergence tolerance. Growth Inhibition: Submergence retarded rice growth, causing leaf yellowing, senescence, and overall plant weakness, with more pronounced effects as submergence duration increased. Significant differences in plant height were observed among seedling cultivation methods and submergence durations. Over two-thirds of tillers survived under complete submergence for up to 7 days during the tillering stage. Submergence significantly affected POD and SOD activities, MDA and PRO contents, and SPAD values, characterized by initial increases followed by decreases in antioxidant enzyme activities, MDA accumulation, and PRO content elevation. Submergence Tolerance Variation: Seedling cultivation methods significantly influenced yield and submergence tolerance. Among treatments, direct seeding showed the poorest submergence tolerance, while hard-ground dry nursery substrate micro-sprinkler tray seedlings combined with machine transplanting performed best. This study demonstrated that rice can retain original plants and maintain a certain yield level under submergence for up to 7 days without special measures, and satisfactory yields can be achieved with appropriate remedial techniques and enhanced management. Seedling cultivation and transplanting methods affect submergence tolerance by regulating plant morphology and physiological adaptability, providing practical insights for optimizing stress-resistant rice cultivation patterns.

## 1 Introduction

Global climate change has spurred a remarkable escalation in extreme weather events. In recent years, flood disasters have witnessed a conspicuous increase in both their scope and frequency when compared to the past. This has frequently led to extensive reductions in crop yields and, in more severe cases, complete crop failures (Pasley et al., 2020; Chadalavada et al., 2021). As a staple crop that is indispensable for global food security, rice is currently facing formidable challenges. Flood stress is pervasive across the globe and takes a heavy toll on the safe production of rice. The majority of rice-growing regions in South Asia and Southeast Asia are subject to the impacts of floods to varying degrees. The Yangtze River Basin in China represents a principal production area for high-quality japonica rice. From late June to late July each year, during the rainy season, rice reaches the peak tillering stage and is highly susceptible to flooding. Such inundation can precipitate severe yield reductions or even wipe out the entire crop, posing a significant menace to rice production. Teams affiliated with the International Rice Research Institute (IRRI) and the Africa Rice Center (Africa-Rice) have achieved a breakthrough by successfully developing rice varieties capable of enduring submerged conditions for two weeks. This innovation not only augments farmers’ economic returns but also bolsters their capacity to grapple with climate change. Consequently, it is of paramount urgency in practical terms and holds crucial strategic significance to conduct more in-depth investigations into the mechanisms underlying rice tolerance to flooding, breed novel flood-tolerant varieties, devise supportive cultivation techniques and management strategies, and furnish robust scientific and technological underpinning to fortify the disaster resistance of the rice industry in the face of climate change challenges (Heredia et al., 2022; Saud et al., 2022).

A wealth of studies has substantiated that when plants are submerged, their incapacity to draw sufficient oxygen from the environment plunges them into an anoxic state, thereby impeding respiration and photosynthesis (Zhou et al., 2020; Bispo and Vieira, 2022). Prolonged flooding stress can cause chlorosis, growth stagnation, and even plant death (Izzawati et al., 2022). No higher plants can tolerate a prolonged anoxic environment. Flooding stress induces hypoxic conditions in the plant’s growth environment, thereby triggering a series of physiological, biochemical, and morphological changes. For instance, a significant increase in antioxidant enzyme activities can, through catalyzing the scavenging of reactive oxygen species, reduce the degree of lipid peroxidation and enhance the plant’s flood tolerance-often describes the ability to withstand the inundation caused by large-scale flooding of rivers, lakes, or other water bodies. Such flooding may cover large areas, including both soil saturation and the complete submersion of plants or buildings in a relatively large region (Izzawati et al., 2022; Liu et al., 2023). The anoxia caused by flooding stress leads to various morphological alterations in rice plants. As the number of submerged days increases, the contents of malondialdehyde (MDA) and proline (PRO) in rice show an upward trend, adversely affecting the integrity of biological membranes, leading to cell membrane damage and causing internal metabolic disorders (Hossain et al., 2009). Under flooding stress, the stomata of rice close, affecting the normal structure of chloroplasts and the synthesis of chlorophyll. Moreover, with the intensification of flooding stress, chlorophyll content decreases significantly, electron transport efficiency declines, and photosynthetic capacity diminishes (Rich et al., 2008; Jethva et al., 2022).

With the advancement of molecular genetics and molecular biology, numerous quantitative trait loci (QTLs) related to rice flood tolerance have been identified; however, most are concentrated in the germination and seedling stages, with few reports in other developmental stages. Moreover, relatively few flood-tolerant QTLs have been finely mapped and cloned. The currently reported and widely applied genes associated with rice flood tolerance mainly include *Submergence1A (Sub1A)* (Alpuerto et al., 2022), *Submergence1B (Sub1B)* (Nishiuchi et al., 2012), *Submergence1C (Sub1C)* (Singh et al., 2020), *Snorkel1 (SK1)* and *Snorkel2 (SK2)* (Nagai et al., 2022), *Calcineurin B-like Protein10 (OsCBL10)*, *UDP-glycosyltransferase75A (OsUGT75A)* (Ye et al., 2018), *OsARD1 (OsARD1-OE)* (Liang et al., 2019), *Trehalose-6-phosphate phosphatase 7 (OsTPP7)* (Aung et al., 2023), among others. These genes enhance rice flood tolerance by regulating metabolic pathways and catalyzing key reactions. *Sub1A* has been extensively studied and proven to play a crucial role in conferring submergence tolerance. The *Sub1* gene is located on chromosome 9 of rice and comprises three ethylene response factor (ERF) transcriptional regulators: *Sub1A*, *Sub1B*, and *Sub1C* (Kaspary and Roma-Burgos, 2020; Haque et al., 2023). Among them, *Sub1A* has two alleles, *Sub1A-1* and *Sub1A-2*. Flood-tolerant rice genotypes contain *Sub1A-1*, while flood-intolerant genotypes contain *Sub1A-2* or lack the *Sub1A-1* gene. Flood tolerance is closely related to the significantly high expression of *Sub1A-1* within 14 days after submergence. The increase in ethylene levels after flooding activates the expression of *Sub1A-1*, and the phenotypic change directly associated with the presence of *Sub1A-1* is the restricted underwater elongation of stems and leaves (Niroula et al., 2012; Winkel et al., 2014; Chakraborty et al., 2021; He et al., 2023; Im and Choi, 2024; Phukan et al., 2023);.

Different methods are highly likely to affect the expression of genes associated with various water - stress tolerances, such as flood and waterlogging tolerance (Nishiuchi et al., 2012). Although flood and waterlogging are different manifestations of water stress, they often trigger similar physiological and genetic responses in plants. Genes involved in adapting to hypoxic conditions, regulating energy metabolism, and maintaining cell integrity play crucial roles in both scenarios. Thus, examples related to waterlogging tolerance can effectively illustrate how cultivation methods influence the overall water - stress tolerance of rice, including flood tolerance. Through the implementation of appropriate cultivation management and technical measures, the flood tolerance of rice can be catalytically enhanced, thereby reducing yield losses. The seedling-raising method significantly impacts the quality of seedlings and their stress resistance. In terms of overall seedling quality, the dry seedling-raising method is superior to the wet method, and the coated dry seedling-raising method is even better than the conventional dry method (Sun et al., 2021). Under relatively low-temperature conditions, hand-transplanted hybrid indica rice will experience total crop failure if submerged for more than 9 days, whereas hand-transplanted, machine-transplanted, and direct-seeded conventional japonica rice will suffer total crop failure if submerged for more than 12 days. From the perspective of different cultivation methods, flood tolerance follows the order: direct-seeded rice > hand-transplanted rice > machine-transplanted rice (Wang et al., 2008). The contents of soluble sugars, starches, total nitrogen, nitrate nitrogen, and nitrate reductase activity in dry-raised seedlings are all higher than those in water-raised or wet-raised seedlings (Ren et al., 2009). In general, different seedling-raising methods have a significant impact on the flood tolerance of rice seedlings. Water-raised and dry-raised seedlings are relatively weak in flood tolerance, while semi-dry-raised seedlings, by integrating the advantages of both methods, improve the flood tolerance of seedlings to a certain extent.

In conclusion, while there have been relevant reports on the physiological, molecular, and cultivation aspects of rice flood tolerance (He et al., 2023; Im and Choi, 2024; Phukan et al., 2023; Sun et al., 2021), studies focusing on the impact of flood stress on the growth and physiological and biochemical characteristics of rice at the tillering stage are relatively scarce. In actual production, there is an urgent need to comprehensively apply various stress-resistant cultivation techniques to catalyze the enhancement of flood tolerance in existing varieties and to optimize their combination according to different ecological environments and planting conditions, thereby improving the flood tolerance and adaptability of rice. Simultaneously, the synergistic effects of these techniques should be studied, and remedial measures after flooding should be explored to mitigate the losses caused by flooding to rice production (Winkel et al., 2014). In this study, Nanjing 46, a variety with a large planting area and excellent quality in the Yangtze River Basin, was used as the experimental material. Different flooding duration treatments were conducted during the tillering stage, and differences in agronomic traits, leaf physiological indicators, and yields under different seedling-raising and cultivation combination methods were compared. The response mechanisms of different seedling-raising and cultivation combinations to flooding stress were deeply explored. The research results cannot only provide a theoretical basis for developing supporting cultivation techniques for flood tolerance and exploring the mechanisms of rice resistance to flood disasters but also offer a practical operational plan for enhancing the disaster resistance of rice cultivation in the Yangtze River Basin and extensive low-lying areas in China (Sun et al., 2021; Khan et al., 2021; Alpuerto et al., 2022; Wang X. et al., 2021).

## 2 Materials and methods

### 2.1 Experimental materials and sites

**Figure 1:**
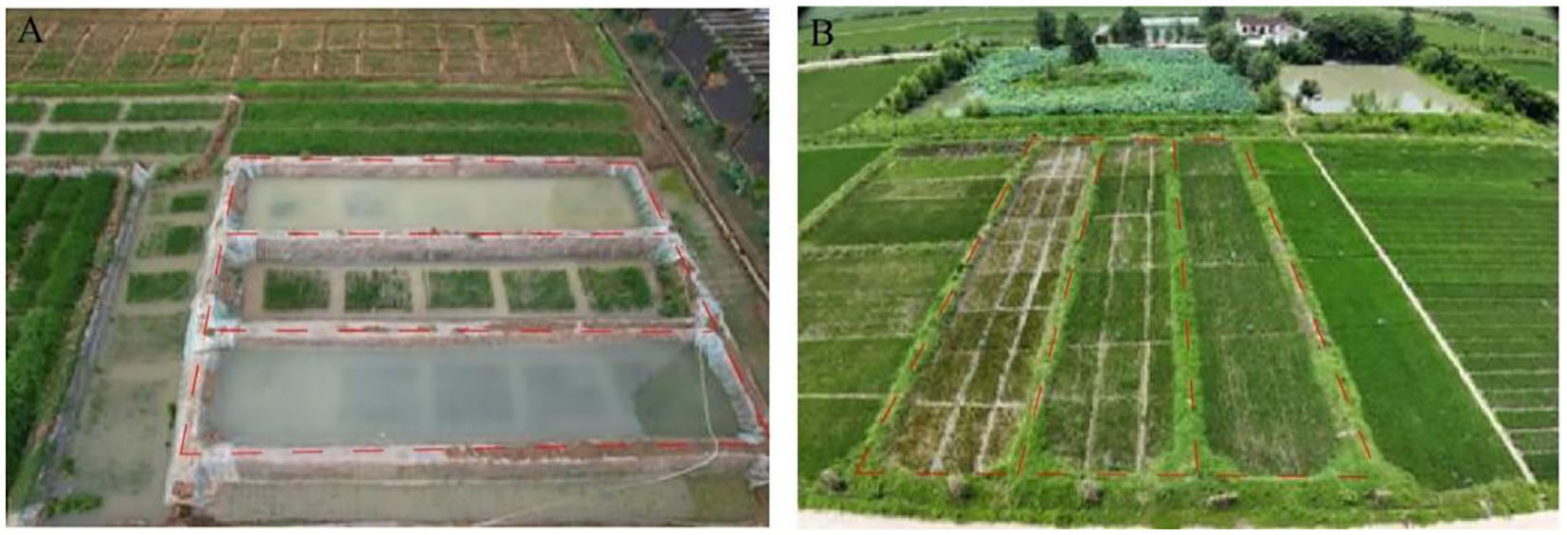
Field planting diagram. **(A)** is an aerial view of the experimental field at Anhui Science and Technology University; **(B)** is an aerial view of the rice experimental base of Anhui Xin Fu Xiang Tian Ecological Agriculture Co., Ltd.

The test variety used in this study was Nanjing 46, a conventional japonica rice cultivar. Experiments were concurrently conducted at two locations: the experimental fields of Anhui Science and Technology University (117°56′E; 32°3′N) and the experimental fields of Anhui Xin Fu Xiang Tian Ecological Agriculture Co., Ltd. (118°18′E; 31°58′N) (**Figure 1**).

### 2.2 Experimental design

The experiment employed a two-factor design. Factor 1 consisted of different seedling cultivation methods: Y1 - artificial dry-bed cultivation, Y2 - artificial moist-bed cultivation, Y3 - conventional nutrient soil cultivation, Y4 - hard-ground dry nursery substrate micro-sprinkler tray cultivation (**Supplementary Material 9**: Technical Key Points of Different Seedling - raising Methods for Y1 - Y4), and Y5 - direct seeding. Factor 2 involved submergence durations: B0 (control) with 0 days of submergence, B1 with 4 days of submergence, B2 with 7 days of submergence, and B3 with 10 days of submergence. Seeds were sown on May 10, with unified seedling management; The seedlings were transplanted at an age of 30 days. According to the local planting habits, direct seeding was carried out on June 7th. The transplanting and sowing density was set at a plant spacing of 15 cm × 20 cm, with three seedlings per hill, and direct seeding was uniformly sown at a rate of 60 kg/hm^2^.

The plot area is 120 square meters. Sampling and data collection were carried out with three replicates within each plot. The submergence treatment was uniformly carried out on the 67th day after transplanting, maintaining a field water level 2–3 cm higher than that of the rice seedlings. Apart from the submergence periods, field water management followed an alternate wetting and drying regime. Basal fertilization was applied 7 days after transplant establishment, with 37.5 kg/hm^2^ of urea and 75 kg/hm^2^ of compound fertilizer. The experimental field was surrounded by a waterproof wall over 1 meter high, lined with waterproof plastic sheeting to prevent leakage. Other water management practices and pest control measures were consistent with standard field production practices.

### 2.3 Measurement indices and methods

Measurements of plant height, number of green leaves, and tiller number for both control and treated plants were conducted one day before submergence and three days after submergence. On the day when the submergence treatment concluded, samples were taken from three points per treatment, with three hills per point to measure the root - to - shoot ratio. For the groups submerged for 7 and 10 days, flag leaves were collected to determine Peroxidase (POD) and Superoxide Dismutase (SOD) activity, Proline (PRO), Malondialdehyde (MDA), and Soil and Plant Analyzer Development (SPAD) values (Mittal et al., 2022).

POD Activity: POD activity was determined using the guaiacol method.

SOD Activity: SOD activity was assayed according to the nitrogen blue tetrazolium (NBT) photoreduction method.

PRO Content: The PRO content was determined using the acid - ninhydrin colorimetric method.

MDA Content: The MDA content was measured using the thiobarbituric acid (TBA) method.

SPAD Values: SPAD values were measured using a SPAD - 502 Plus chlorophyll meter (Konica Minolta, Japan). For each sample, at least 10 fully expanded leaves were selected, and the SPAD values were measured at three different positions on each leaf. The average value of all measurements was considered as the SPAD value for that sample.

At the maturity stage, samples were collected for indoor analysis of panicle traits, actual yield, and theoretical yield. Comprehensive evaluations of flood tolerance were performed using principal component analysis and membership function method. The flood - tolerance coefficient was analyzed.


$$\text{The plant height growth coefficient }=\frac{\text{Control plant height on the survey day}}{\text{Control plant height on the initial day}}$$

$$\text{Relative plant height change rate (\%)}=\frac{\text{Post - submergence plant height}}{\text{Pre - submergence plant height}\times \text{Plant height growth coefficient}}$$

$$\text{Tiller mortality rate }(\%)=\frac{\text{Dead tillers within three days after submergence}}{\text{The number of tillers before submergence}}\times 100$$

$$\text{Tiller growth rate }(\%)=\frac{\text{(Tillers 50 days after submergence}-\text{Tillers before submergence)}}{\text{(Tillers before submergence}\times \text{Tiller increase coefficient)}}\times 100$$

$$\text{The tiller increase coefficient}=\frac{\text{Control tiller number on the survey day}}{\text{Control tiller number on the initial day}}$$

$$\text{Leaf growth coefficient }=\frac{\text{Control green leaf number on the survey day}}{\text{Control green leaf number on the initial day}}$$

$$\text{Relative leaf survival rate}(\%)=\frac{\text{Post-submergence green leaf number}}{\text{(Pre-submergence green leaf number}\times \text{Leaf growth coefficient)}}$$

$$\text{Submergence tolerance index}=\frac{\text{Measured value under submergence treatment}}{\text{Measured value of normal water management}}$$

### 2.4 Data processing

Data were processed using Excel, charts were plotted using Origin 2019, and statistical analysis was performed using SPSS 20.0.One-way analysis of variance was used to evaluate the significant differences among treatments, and the least significant difference (LSD) method was employed for multiple comparisons. Different lowercase letters were used to indicate that the significant difference level reached P< 0.05.

## 3 Results

### 3.1 Effects of different submergence durations on growth period

Submergence led to a consistent delay in the maturity period of rice across all five seedling cultivation methods compared with the control group (**Tables 1**–**4**). The extent of the delay became more pronounced as the number of submergence days increased. After 4 days of submergence, the maturity periods of the different seedling cultivation treatments were delayed by 3 to 11 days relative to the non-submerged control. Specifically, Y3 exhibited the shortest delay in the entire growth period, while Y5 showed the longest delay. Following 7 days of submergence, the delay for each treatment increased to 6 to 15 days, with Y2 having the shortest delay and Y5 still exhibiting the longest delay in the entire growth period. After 10 days of submergence, the delay in the maturity period for each treatment extended to 15 to 25 days. At this point, Y3 experienced the greatest delay in the entire growth period, while Y1 had the shortest delay.

**Table 1:** Differences in growth periods across treatments (B0).

Treatment	Sowing	Heading date	Full heading date	Maturity date	Total growth (d)
Y1	5/10	9/2	9/11	10/21	164
Y2	5/10	9/3	9/9	10/25	168
Y3	5/10	9/4	9/12	10/24	167
Y4	5/10	9/6	9/14	10/27	169
Y5	6/7	9/5	9/8	11/9	155

Remarks: Initial Heading date: When 10% of the rice in the field starts to head, it is the initial heading date; Full heading date: When 80% of the rice panicles in the field are out, it is the full heading date; Maturity date: When 90% of the rice glumes in the field turn yellow, it is the maturity date; Y1, Dry nursery seedlings + manual transplanting; Y2, Wet nursery seedlings + manual transplanting; Y3, Nutrient soil nursery seedlings + mechanical transplanting; Y4, Hard ground substrate micro - sprinkler nursery seedlings + mechanical transplanting; Y5, Direct seeding; B0, Submerged for 0 days (CK).

**Table 2:** Differences in growth periods across treatments (B1).

Treatment	Sowing	Heading date	Full heading date	Maturity date	Total growth (d)
Y1	5/10	9/7	9/15	10/28	171
Y2	5/10	9/7	9/14	11/3	174
Y3	5/10	9/9	9/16	11/7	181
Y4	5/10	9/11	9/19	11/8	182
Y5	6/7	9/11	9/14	11/24	170

Remarks: Initial Heading date: When 10% of the rice in the field starts to head, it is the initial heading date; Full heading date: When 80% of the rice panicles in the field are out, it is the full heading date; Maturity date: When 90% of the rice glumes in the field turn yellow, it is the maturity date; Y1, Dry nursery seedlings + manual transplanting; Y2, Wet nursery seedlings + manual transplanting; Y3, Nutrient soil nursery seedlings + mechanical transplanting; Y4, Hard ground substrate micro - sprinkler nursery seedlings + mechanical transplanting; Y5, Direct seeding; B1, submerged for 4 days.

**Table 3:** Differences in growth periods across treatments (B2).

Treatment	Sowing	Heading date	Full heading date	Maturity date	Total growth (d)
Y1	5/10	9/3	9/12	10/26	169
Y2	5/10	9/5	9/13	10/29	172
Y3	5/10	9/7	9/14	10/30	170
Y4	5/10	9/8	9/17	11/5	176
Y5	6/7	9/9	9/12	11/20	166

Remarks: Initial Heading date: When 10% of the rice in the field starts to head, it is the initial heading date; Full heading date: When 80% of the rice panicles in the field are out, it is the full heading date; Maturity date: When 90% of the rice glumes in the field turn yellow, it is the maturity date; Y1, Dry nursery seedlings + manual transplanting; Y2, Wet nursery seedlings + manual transplanting; Y3, Nutrient soil nursery seedlings + mechanical transplanting;Y4, Hard ground substrate micro - sprinkler nursery seedlings + mechanical transplanting; Y5, Direct seeding; B2, submerged for 7 days.

**Table 4:** Differences in growth periods across treatments (B3).

Treatment	Sowing	Heading date	Full heading date	Maturity date	Total growth (d)
Y1	5/10	9/11	9/20	11/5	179
Y2	5/10	9/9	9/19	11/11	185
Y3	5/10	9/11	9/21	11/19	192
Y4	5/10	9/14	9/23	11/14	188
Y5	6/7	9/16	9/21	11/27	173

Remarks: Initial Heading date: When 10% of the rice in the field starts to head, it is the initial heading date; Full heading date: When 80% of the rice panicles in the field are out, it is the full heading date; Maturity date: When 90% of the rice glumes in the field turn yellow, it is the maturity date; Y1, Dry nursery seedlings + manual transplanting; Y2, Wet nursery seedlings + manual transplanting; Y3, Nutrient soil nursery seedlings + mechanical transplanting; Y4, Hard ground substrate micro - sprinkler nursery seedlings + mechanical transplanting; Y5, Direct seeding; B3, submerged for 10 days.

### 3.2 Effects of submergence treatment on rice plant morphology under different cultivation methods

**Figure 2:**
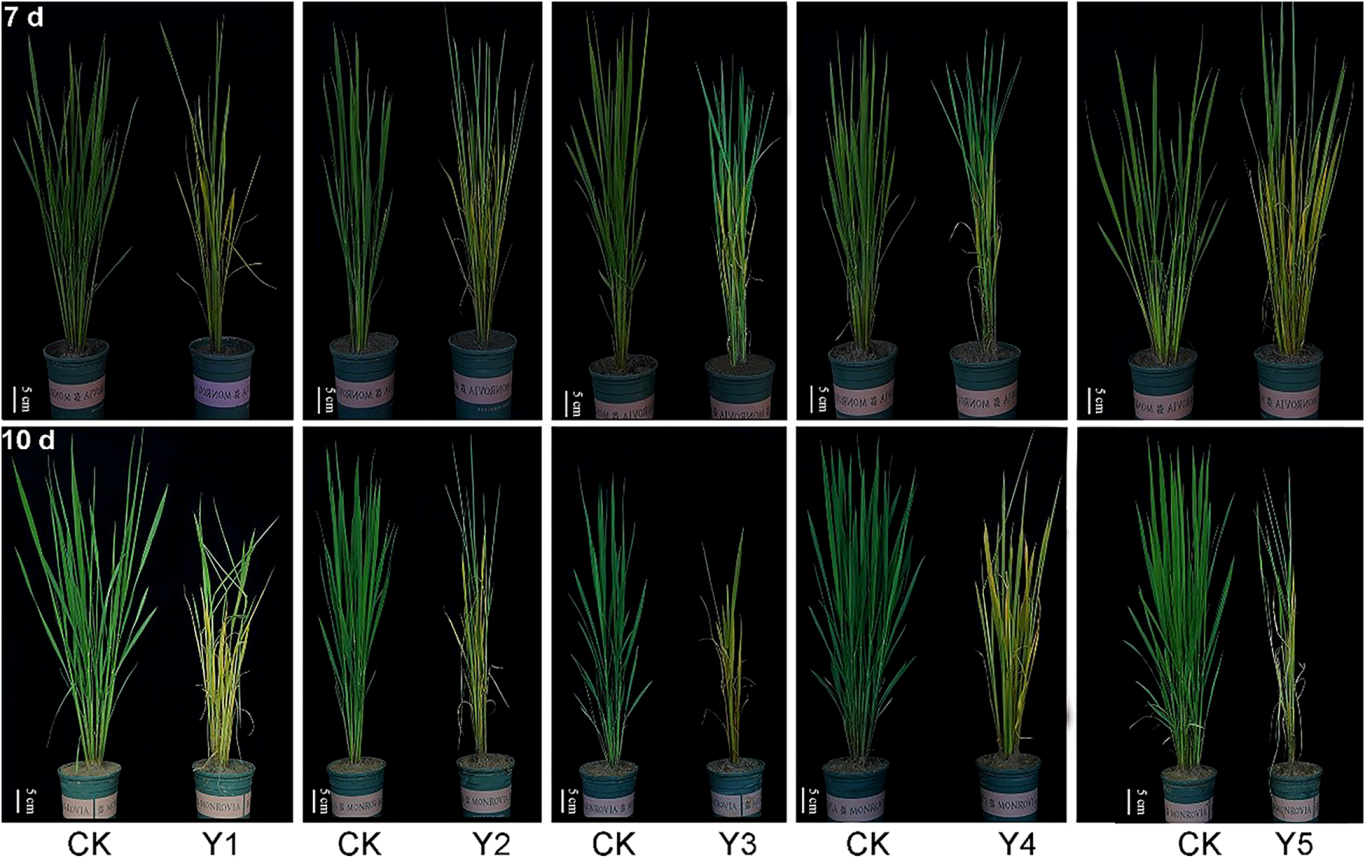
Morphological images of rice under different cultivation methods and submergence durations (Top: 7 days; Bottom: 10 days). Scale bar: 5 cm; Y1 - artificial dry-bed cultivation, Y2 - artificial moist-bed cultivation, Y3 - conventional nutrient soil cultivation, Y4 - hard-ground dry nursery substrate micro-sprinkler tray cultivation, and Y5 - direct seeding; CK represent the photos of blank control plants without submergence treatment. A1, B1, C1, D1, E1 respectively correspond to the comparison photos of the seedlings of five seedling - raising methods Y1, Y2, Y3, Y4, Y5 after being submerged for 7 days; A2, B2, C2, D2, E2 respectively correspond to the comparison photos of the seedlings of five seedling - raising methods Y1, Y2, Y3, Y4, Y5 after being submerged for 10 days; The length of the scale is 5 centimeters.

As illustrated in **Figure 2**, submergence treatment during the tillering stage led to pronounced yellowing of leaves, senescence of older leaves, and an overall frail and weakened plant morphology compared to the control (Alpuerto et al., 2022). After 7 days of submergence, the central leaves of most plants remained viable. However, when the submergence period extended to 10 days, the severity of damage symptoms escalated markedly. The plants exhibited intensified chlorosis, leaf necrosis, and significant stunting. Particularly in the Y3 conventional nutrient soil cultivation and Y5 direct seeding methods, only the central leaves of the main stem survived, while other parts of the plants showed severe stress symptoms and substantial mortality. This highlights the differential impact of submergence stress across various cultivation methods, with conventional nutrient soil and direct seeding methods being more susceptible to prolonged submergence.

### 3.3 Effects of submergence treatment on agronomic traits of rice under different seedling cultivation methods

The plant height results at maturity for each treatment are shown in **Table 5**. From **Table 5**, it can be seen that submergence for up to 7 days had a minimal impact on rice plant height. However, when the submergence duration extended to 10 days, there was a significant reduction in plant height compared to the control. Notably, the Y3 nutrient soil seedling cultivation treatment showed the greatest reduction in plant height, with a decrease of 22.84 cm compared to the control. This indicates that prolonged submergence significantly inhibits rice growth, and different seedling cultivation methods exhibit varying levels of tolerance to submergence. There were highly significant differences in plant height between different seedling cultivation methods and submergence durations, highlighting the importance of seedling cultivation methods and submergence time on rice plant height. By integrating appropriate seedling management practices in actual production, it is possible to better cope with varying degrees of submergence stress, enhancing rice resilience and yield stability.

**Table 5:** Effects of different treatments on plant height at maturity (cm).

Treatment	B0	B1	B2	B3
Y1	91.33 ± 2.75*b*	97.00 ± 6.50*b*	100.00 ± 3.00*a*	88.33 ± 2.89*a*
Y2	97.83 ± 3.33*a*	102.5 ± 9.84*a*	99.83 ± 2.75*a*	78.23 ± 3.76*b*
Y3	98.67 ± 3.06*a*	96.00 ± 1.73*b*	101.00 ± 1.73*a*	75.83 ± 1.04*b*
Y4	98.83 ± 2.93*a*	95.83 ± 2.84*b*	94.33 ± 3.06*b*	80.07 ± 1.32*b*
Y5	83.50 ± 5.2*c*	90.67 ± 1.26*c*	99.00 ± 3.61*a*	79.88 ± 1.27*b*

Remark: Y1 is dry nursery seedlings + manual transplanting; Y2 is wet nursery seedlings + manual transplanting; Y3 is nutrient soil nursery seedlings + mechanical transplanting; Y4 is hard ground substrate micro - sprinkler nursery seedlings + mechanical transplanting; Y5 is direct seeding; B0 is submerged for 0 days (CK); B1 is submerged for 4 days; B2 is submerged for 7 days; B3 is submerged for 10 days. One-way analysis of variance (ANOVA) and the least significant difference (LSD) method were used for multiple comparisons. Different lowercase letters indicate significant differences among treatments (*P*< 0.05).

The number of dead leaves and sum of leaf numbers were recorded on the day of submergence treatment, and the survival rate of green leaves was calculated. As shown in **Table 6**, submergence for a relatively short duration of 4 days resulted in a green leaf survival rate above 80% across different seedling cultivation methods. However, when the submergence duration reached 7 days, the green leaf survival rates for Y4 (hard substrate seedling cultivation) and Y5 (direct seeding) dropped to 78.10% and 68.1%, respectively. With 10 days of submergence, the green leaf survival rate declined even more markedly, with direct seeding showing a survival rate of only 41.00%. The dry-bed and moist-bed seedling cultivation methods demonstrated a relative advantage in maintaining higher green leaf survival rates under prolonged submergence conditions.

**Table 6:** Effects of different treatments on green leaf survival rate (%).

Treatment	B1	B2	B3
Y1	93.44 ± 0.03*a*	87.40 ± 0.03*a*	65.40 ± 0.09*a*
Y2	83.4 ± 0.03*bc*	82.10 ± 0.04*ab*	68.00 ± 0.09*a*
Y3	85.5 ± 0.04*b*	84.60 ± 0.05*a*	47.20 ± 0.13*b*
Y4	81.9 ± 0.03*c*	78.10 ± 0.08*b*	49.30 ± 0.09*bc*
Y5	82.9 ± 0.08*bc*	68.10 ± 0.11*c*	41.00 ± 0.05*c*

Remark: Y1 is dry nursery seedlings + manual transplanting, Y2 is wet nursery seedlings + manual transplanting, Y3 is nutrient soil nursery seedlings + mechanical transplanting, Y4 is hard ground substrate micro-sprinkler nursery seedlings + mechanical transplanting, Y5 is direct seeding; B1 is submerged for 4 days, B2 is submerged for 7 days, B3 is submerged for 10 days; One-way analysis of variance (ANOVA) and the least significant difference (LSD) method were used for multiple comparisons. Different lowercase letters indicate significant differences among treatments (*P*< 0.05).

As shown in **Table 7**, the duration of submergence during the rice tillering stage significantly impacted the number of tillers. Submergence for 4 days did not result in a significant difference in tiller numbers compared to the control. However, submergence beyond 7 days led to a noticeable decrease in the number of tillers. Specifically, the Y3 nutrient soil seedling cultivation showed a 53.33% reduction in tiller number after 10 days of submergence compared to the control, and the Y5 direct seeding method left primarily the main stem. Within the 7-day submergence period, the number of tillers among different seedling cultivation methods did not show significant differences, except for the direct seeding method, which differed significantly from the other methods. When submergence reached 10 days, the tiller numbers in Y3 nutrient soil seedling cultivation showed significant differences compared to Y1 dry-bed cultivation and Y2 moist-bed cultivation. As the submergence duration increased, the survival rate of rice tillers decreased rapidly. However, within 7 days of complete submergence during the tillering stage, more than two-thirds of the tillers remained viable, indicating that rice submerged for up to 7 days during the tillering stage still has potential value.

**Table 7:** Effects of submergence on the number of tillers under different seedling cultivation methods (tillers/hill).

Treatment	CK-B1	B1	CK-B2	B2	CK-B3	B3
Y1	10.67 ± 1.53*ab*	10.00 ± 2.00*ab*	11.00 ± 1.00*c*	10.33 ± 1.53*a*	11.33 ± 1.15*c*	9.33 ± 1.15*a*
Y2	10.33 ± 2.08*ab*	9.67 ± 2.52*ab*	12.33*b* ± 2.31*c*	11.67 ± 2.08*a*	12.33 ± 2.31*bc*	9.33 ± 1.53*a*
Y3	11.67 ± 2.52*a*	10.67 ± 2.08*ab*	14.33 ± 3.21*ab*	10.33 ± 1.53*a*	15.00 ± 1.00*ab*	7.00 ± 1.00*b*
Y4	13.67 ± 9.29*a*	13.33 ± 9.45*a*	16.33 ± 2.31*a*	11.00 ± 1.73*a*	16.33 ± 4.16*a*	8.33 ± 3.21*ab*
Y5	3.00 ± 0.00*b*	1.33 ± 0.58*b*	3.00 ± 0.00*d*	1.3 ± 0.58*b*	3.00 ± 0.00*d*	1.33 ± 0.58*c*

Remark: Y1 is dry nursery seedlings + manual transplanting; Y2 is wet nursery seedlings + manual transplanting; Y3 is nutrient soil nursery seedlings + mechanical transplanting; Y4 is hard ground substrate micro - sprinkler nursery seedlings + mechanical transplanting; Y5 is direct seeding; B1 is submerged for 4 days; CK - B1 represents the control group for B1; B2 is submerged for 7 days; CK - B2 represents the control group for B2; B3 is submerged for 10 days; CK - B3 represents the control group for B3. One-way analysis of variance (ANOVA) and the least significant difference (LSD) method were used for multiple comparisons. Different lowercase letters indicate significant differences among treatments (*P*< 0.05).

As shown in **Table 8**, the root-to-shoot ratio exhibited an increasing trend with prolonged submergence duration. After 4 days of submergence, the root-to-shoot ratio for Y4 hard substrate seedling cultivation was outstanding, showing significant differences compared to other seedling cultivation methods. After 7 days of submergence, the differences in root-to-shoot ratios among treatments were not significant. However, following 10 days of submergence, both Y4 hard substrate seedling cultivation and Y3 nutrient soil seedling cultivation displayed higher root-to-shoot ratios, with Y4 hard substrate seedling cultivation showing significant differences from the other treatments.

**Table 8:** Effects of submergence on the root-to-shoot ratio under different seedling cultivation methods (root-to-shoot ratio).

Treatment	B0	CK-B1	B1	CK-B2	B2	CK-B3	B3
Y1	0.19 ± 0.02*a*	0.19 ± 0.02*a*	0.22 ± 0.02*c*	0.15 ± 0.01*b*	0.28 ± 0.06*a*	0.18 ± 0.02*b*	0.18 ± 0.03*b*
Y2	0.14 ± 0.03*a*	0.17 ± 0.02*a*	0.21 ± 0.02*c*	0.13 ± 0.02*b*	0.32 ± 0.09*a*	0.14 ± 0.02*bc*	0.18 ± 0.06*b*
Y3	0.16 ± 0.02*a*	0.19 ± 0.01*a*	0.26 ± 0.01*bc*	0.16 ± 0.01*b*	0.30 ± 0.06*a*	0.19 ± 0.01*ab*	0.31 ± 0.01*bc*
Y4	0.20 ± 0.02*a*	0.19 ± 0.02*a*	0.36 ± 0.05*a*	0.29 ± 0.02*a*	0.40 ± 0.17*a*	0.28 ± 0.02*a*	0.42 ± 0.07*a*
Y5	0.12 ± 0.02*b*	0.12 ± 0.02*b*	0.29 ± 0.01*b*	0.11 ± 0.05*c*	0.32 ± 0.03*a*	0.12 ± 0.03*c*	0.22 ± 0.04*b*

Remark: Y1 is dry nursery seedlings + manual transplanting, Y2 is wet nursery seedlings + manual transplanting, Y3 is nutrient soil nursery seedlings + mechanical transplanting, Y4 is hard ground substrate micro - sprinkler nursery seedlings + mechanical transplanting, Y5 is direct seeding; B0 is submerged for 0 days (CK), B1 is submerged for 4 days, CK - B1 represents the control group for B1; B2 is submerged for 7 days, CK - B2 represents the control group for B2; B3 is submerged for 10 days, CK - B3 represents the control group for B3; One-way analysis of variance (ANOVA) and the least significant difference (LSD) method were used for multiple comparisons. Different lowercase letters indicate significant differences among treatments (*P*< 0.05).

As shown in **Supplementary Tables S1**-**S3**, the relative change rates of plant height compared to the control were relatively minor, with Y1 dry-bed cultivation and Y5 direct seeding both exhibiting values greater than 1, indicating an increasing trend. After 7 days of submergence, the relative increase in plant height reached 20%. Submergence significantly reduced the relative survival rate of green leaves, which plummeted with prolonged submergence duration. After 10 days of submergence, the relative survival rate of green leaves in Y5 direct seeding dropped to only 24.00%. For relatively short submergence durations (4 days), the tiller mortality rate was highest in Y5 direct seeding, followed by Y3 nutrient soil cultivation, with no significant differences among the various seedling methods. As the submergence duration increased, significant differences emerged among the different seedling methods; after 10 days of submergence, the tiller mortality rates in Y5 direct seeding and Y2 moist-bed cultivation soared to 61%. Upon conclusion of the submergence treatment and after 50 days of recovery growth, the tiller growth rate was measured. Within the 7-day submergence treatments, Y1 dry-bed cultivation and Y4 hard substrate seedling cultivation exhibited positive growth rates. However, with 10 days of submergence, all treatments showed negative growth rates, indicating a downward trend compared to the control, with the most pronounced decrease observed in Y3 nutrient soil seedling cultivation.

### 3.4 Effects of submergence treatment on leaf physiological indices of rice under different cultivation methods

**Figure 3:**
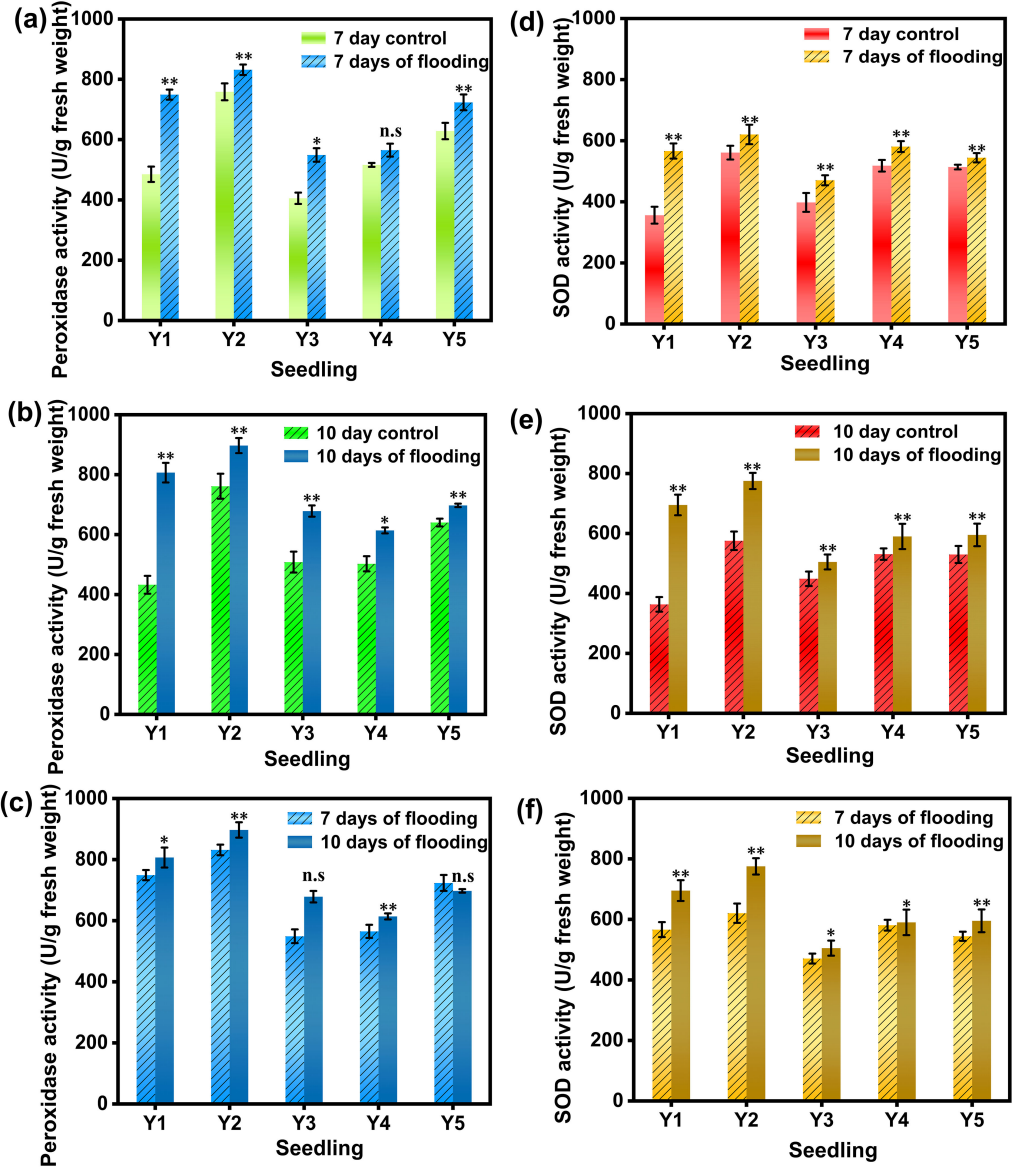
POD activity and SOD activity in rice leaves under different treatments. **(a)** shows the POD activity values of the leaves of the group flooded for 7 days and the non-flooded control group; **(b)** shows the POD activity values of the leaves of the group flooded for 10 days and the non-flooded control group; **(c)** shows the POD activity values of the leaves of the group flooded for 7 days and the control group for the 10 - day flooded group; **(d)** shows the SOD activity values of the leaves of the group flooded for 7 days and the non-flooded control group; **(e)** shows the SOD activity values of the leaves of the group flooded for 10 days and the non-flooded control group; **(f)** shows the SOD activity values of the leaves of the group flooded for 7 days and the control group corresponding to the 10 - day flooded group;Y1 is dry nursery seedlings + manual transplanting, Y2 is wet nursery seedlings + manual transplanting, Y3 is nutrient soil nursery seedlings + mechanical transplanting, Y4 is hard ground substrate micro - sprinkler nursery seedlings + mechanical transplanting, Y5 is direct seeding. The asterisk (*) indicates a significant difference, typically corresponding to P<0.05; double asterisks (**) indicate a highly significant difference, usually corresponding to P<0.01; and "n.s." denotes no significant difference.

Peroxidase (POD) is a class of enzymes that catalyze the oxidation of substrates using hydrogen peroxide as an electron acceptor. It plays a crucial role in the antioxidant defense system within organisms. Peroxidase can catalyze the oxidation reaction of hydrogen peroxide with various substrates. For example, in plants, it is involved in processes such as photosynthesis, respiration, cell wall synthesis and lignification, and is also related to plant stress resistance. As shown in **Figure 3**, after 7 days of submergence, significant differences in POD activity were observed between the control and the other four seedling cultivation methods, except for Y4 hard substrate micro-sprinkler cultivation. Among them, the POD activity of moist-bed cultivation was 1.54 times that of the control (non-submerged) (**Figure 3a**). After 10 days of submergence, the POD activity in the five different seedling cultivation methods increased by 86.4%, 17.8%, 33.5%, 22.1%, and 8.9%, respectively, compared to the control, with all showing significant differences (**Figure 3b**). At 10 days of submergence, the POD activity in the direct seeding method showed a decreasing trend, while the other four cultivation methods exhibited increased POD activity compared to 7 days of submergence, with the nutrient soil cultivation method showing an increase of up to 18.2% (**Figure 3c**).

Superoxide Dismutase (SOD) is a metal - containing enzyme widely present in organisms. It can catalyze the disproportionation reaction of superoxide anion radicals and convert them into oxygen and hydrogen peroxide. Thus, it removes superoxide anion radicals from organisms and plays a crucial role in maintaining the intracellular redox balance and normal cell functions. As shown in **Figure 3d-f**, after 7 days of submergence, the SOD activity in rice leaves under the five different seedling cultivation methods was 1.58, 1.11, 1.18, 1.12, and 1.06 times that of the control (non-submerged), respectively. Notably, water stress significantly increased the SOD activity in rice cultivated using the moist-bed method, with all five submergence treatment groups showing significant differences from the control (**Figure 3d**). After 10 days of submergence, the SOD activity in the moist-bed and dry-bed cultivation methods showed a marked increase compared to the control, with the moist-bed method exhibiting the highest increase of 56.6%. Significant differences in SOD activity were observed between the five submergence treatments and the control (**Figure 3e**). The SOD activity in rice leaves across all five cultivation methods demonstrated an increasing trend with prolonged submergence duration. This indicates that submergence stress enhances the activity of antioxidative enzymes in leaves, and this enhancement becomes more pronounced with longer submergence periods, thereby improving the rice plants’ ability to scavenge reactive oxygen species (ROS) (**Figure 3f**) (Wang W. et al., 2021).

**Figure 4:**
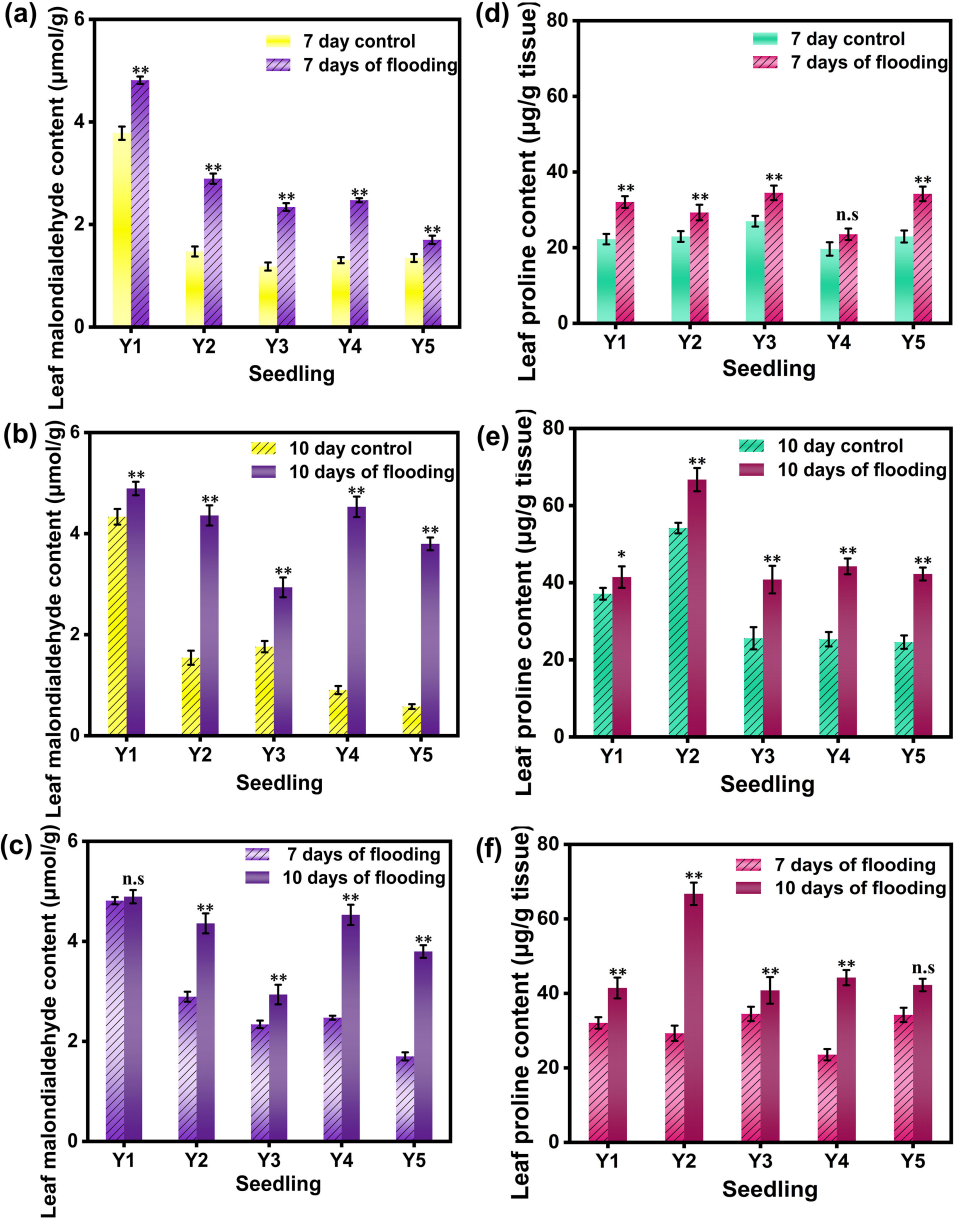
MDA content and PRO content in rice leaves under different treatments. **(a)** shows the MDA activity values of the leaves of the flooded for 7 days group and the non -flooded control group; **(b)** shows the MDA activity values of the leaves of the flooded for 10 days group and the non -flooded control group; **(c)** shows the MDA activity values of the leaves of the flooded for 7 days group and 10 days control group; **(d)** shows the PRO activity values of the leaves of the flooded for 7 days group and the non -flooded control group; **(e)** shows the PRO activity values of the leaves of the flooded for 10 days group and the non -flooded control group; **(f)** shows the PRO activity values of the leaves of the flooded for 7 days group and 10 days control group. Y1 is dry nursery seedlings + manual transplanting; Y2 is wet nursery seedlings + manual transplanting; Y3 is nutrient soil nursery seedlings + mechanical transplanting; Y4 is hard ground substrate micro - sprinkler nursery seedlings + mechanical transplanting; Y5 is direct seeding. The asterisk (*) indicates a significant difference, typically corresponding to P<0.05; double asterisks (**) indicate a highly significant difference, usually corresponding to P<0.01; and "n.s." denotes no significant difference.

Malondialdehyde (MDA) is a critical indicator of the degree of lipid peroxidation in plant cell membranes. Elevated MDA content signifies a higher extent of lipid peroxidation, indicating damage to the cell membrane (Mittal et al., 2022). The MDA content in rice leaves was measured before and after submergence treatment (see **Figure 4**). After 10 days of submergence, the accumulation and rate of increase of MDA in rice leaves under the five different cultivation methods were significantly higher than those observed after 7 days of submergence. This indicates that the degree of lipid peroxidation in cell membranes was greater after 10 days of submergence than after 7 days, and it intensified with prolonged submergence stress. This finding aligns with the observation that antioxidative enzyme activity in rice significantly increased post-submergence treatment compared to the control. It is hypothesized that the initial phase of submergence stress triggers an enhanced antioxidative response in rice plants, which escalates as the stress intensity increases. After 10 days of submergence, the accumulation and rate of increase of MDA in rice leaves were significantly higher under four out of the five different cultivation methods compared to those after 7 days of submergence, with the wet nursery method being the exception. There were significant differences in MDA content between the 7-day and 10-day submergence treatments compared to the control, with MDA content being higher in the 10-day submergence treatment than in the 7-day treatment.

Proline (PRO) is an important osmotic regulator that plays a crucial role in physiological processes such as respiration (Thanwisai et al., 2022; Shrestha et al., 2021). The accumulation of PRO can serve as an indicator of the stress level endured by rice plants. By monitoring the changes in proline concentration over time, researchers can gain in-depth insights into the severity of the submergence impact on the plants and the effectiveness of their stress response. This information is of great value for understanding the stress response mechanisms of plants and formulating strategies to enhance their tolerance in future agricultural practices. Under submergence stress, the PRO content in rice leaves varies significantly among different seedling cultivation methods. As the duration of submergence stress increases, the PRO content in rice leaves under five different cultivation methods significantly increases compared to the control. After 7 days of submergence, the increases in PRO content in rice leaves for the five cultivation methods were 44.05%, 27.55%, 27.78%, 19.76%, and 49.04%, respectively. Except for the hard substrate micro - sprinkler cultivation method, which showed no significant difference from the control, the other four cultivation methods exhibited significant differences in PRO content compared to the control. After 10 days of submergence, the PRO content in rice leaves under all five cultivation methods showed significant differences compared to the control, with the highest increase observed in the dry seedling cultivation method. Compared to the 7 - day submergence treatment, the 10 - day submergence treatment showed significant differences in PRO content for all cultivation methods except for the direct seeding method. These results indicate that under submergence stress during the tillering stage, rice leaves can maintain a high level of osmotic regulators as the duration of submergence increases. The dry seedling cultivation method enhances the osmotic regulation in rice leaves, effectively reducing the damage caused by submergence stress.

**Figure 5:**
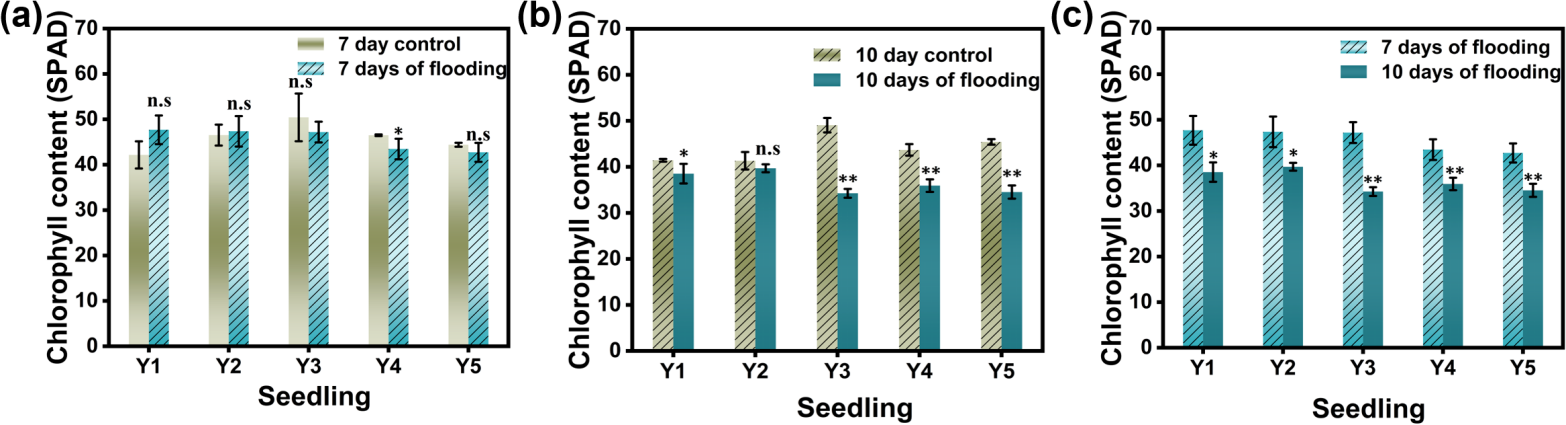
SPAD values of rice leaves under different treatments. **(a)** shows the SPAD activity values of the leaves of the flooded for 7 days group and the non -flooded control group, **(b)** shows the SPAD activity values of the leaves of the flooded for 10 days group and the non -flooded control group;**(c)** shows the SPAD activity values of the leaves of the flooded for 7 days group and 10 days control group; Y1 is dry nursery seedlings + manual transplanting, Y2 is wet nursery seedlings + manual transplanting, Y3 is nutrient soil nursery seedlings + mechanical transplanting, Y4 is hard ground substrate micro - sprinkler nursery seedlings + mechanical transplanting, Y5 is direct seeding. The asterisk (*) indicates a significant difference, typically corresponding to P<0.05; double asterisks (**) indicate a highly significant difference, usually corresponding to P<0.01; and "n.s." denotes no significant difference.

As shown in **Figure 5**, submergence has a certain impact on the SPAD values of rice leaves. After 7 days of submergence, the SPAD values of leaves in Y1 (dry seedling cultivation) and Y2 (wet seedling cultivation) increased compared to the control, while the SPAD values of leaves in Y3 (nutrient soil seedling cultivation), Y4 (hard substrate seedling cultivation), and Y5 (direct seeding) were lower than the control. After 10 days of submergence, the SPAD values of rice leaves in all five cultivation methods were lower than the control. There were significant differences in SPAD values between different seedling cultivation methods under control conditions, but these differences were not significant after submergence treatment.

### 3.5 Impact of submergence treatment on rice yield under different cultivation methods

**Supplementary Tables S4**-**S7** indicate that different submergence durations and seedling cultivation methods significantly influence multiple rice yield components, such as effective panicles per hill, grains per panicle, filled grains per panicle, seed - setting rate, 1000 - grain weight, aboveground biomass, yield, and harvest index. Tillering - stage submergence stress doesn’t notably affect panicle length or seed - setting rate but significantly impacts effective panicle number, grains per panicle, 1000 - grain weight, and actual yield. It reduces these components, and the longer the stress, the greater the impact, ultimately causing yield loss.

The interaction between cultivation methods and submergence duration significantly affects effective panicles, panicle length, aboveground biomass, and 1000-grain weight, but has no significant impact on filled grains per panicle, seed setting rate, harvest index, and yield. The treatments with Y4 hard substrate cultivation and Y2 wet cultivation show higher filled grain numbers and seed setting rates under both control and submergence conditions, resulting in higher theoretical and actual yields and demonstrating relatively stronger submergence tolerance.

The F-values for blocks, treatments, cultivation methods (Y), submergence durations (B), and their interactions all show highly significant differences. As the duration of submergence increases, the number of effective panicles per hill decreases. Within the same seedling cultivation method, the change in panicle length is not significant; however, there are significant differences in other yield components. When comparing the same seedling cultivation method, the differences between the control and the 4-day submergence treatment are not significant. However, with submergence lasting 7 days or more, the differences become increasingly pronounced. After 10 days of submergence, the differences among various yield components are extremely significant.

Yield is a crucial indicator for assessing rice growth, directly reflecting the extent of damage caused by submergence treatment. The yield reduction rate is also a relatively direct metric for evaluating rice’s tolerance to submergence ^[33].^ From both theoretical and actual yield perspectives, the Y4 hard substrate seedling cultivation and Y2 wet seedling cultivation treatments exhibit higher yields in both the control and waterlogged groups. Regarding yield reduction caused by submergence, a 4-day submergence duration has minimal impact on actual yield, with the Y5 direct-seeding treatment experiencing the highest reduction rate of 19.42%. The Y2 andY4 seedling cultivation methods exhibit yield reductions within 10%. When the submergence duration reaches 7 days, the most significant reduction in actual yield is observed in the Y5 direct-seeding method, with a decrease of 35.45%. After 10 days of submergence, the most substantial yield reduction remains in the Y5 direct-seeding method, with a decrease of 56.65%. Even after 7 days of submergence, although the rice yield is significantly lower than the control, the Y1 dry seedling cultivation method (except for the Y5 direct-seeding method) still achieves a yield of 6594.04 kg/hm² (**Table 9**). This indicates that when rice is submerged for up to 7 days during the tillering stage, retaining the original plants can still result in a reasonable yield, even without special remedial measures. If appropriate remedial cultivation measures and enhanced management are implemented later, it is still possible to achieve a satisfactory yield ^[34]^. Additionally, comparing theoretical and actual yields, the overall trend of the impact on yield by different seedling cultivation methods and different submergence durations is consistent (**Table 10**).

**Table 9:** Impact of submergence treatment on actual yield, including block actual yield (kg/hm^2^) and ± B0(%).

Treatment	Actual yield (kg/hm^2^)				± B0 (%)		
	B0	B1	B2	B3	B1	B2	B3
Y1	8662.66 ± 681.73*ab*	7507.46 ± 1514.01*a*	6594.04 ± 794.86*ab*	4945.99 ± 1109.43*b*	-13.34	-23.88	-42.9
Y2	9519.57 ± 2836.92*a*	8881.29 ± 3005.44*a*	7724.23 ± 1219.41*a*	5619.48 ± 1289.13*b*	-6.7	-18.86	-40.97
Y3	8464.42 ± 2130.95*ab*	7614.92 ± 712.23*a*	6911.79 ± 1006.62*a*	5966.87 ± 1289.12*a*	-10.04	-18.34	-29.51
Y4	9534.39 ± 111.89*a*	9278.71 ± 4390.44*a*	8255.98 ± 778.25*a*	7028.51 ± 408.15*a*	-2.68	-13.41	-26.28
Y5	6569.02 ± 1304.62*b*	5292.46 ± 1403.28*b*	4240.08 ± 1406.75*b*	2847.72 ± 713.02*b*	-19.43	-35.45	-56.65

Remark: Y1 is dry nursery seedlings + manual transplanting; Y2 is wet nursery seedlings + manual transplanting; Y3 is nutrient soil nursery seedlings + mechanical transplanting; Y4 is hard ground substrate micro - sprinkler nursery seedlings + mechanical transplanting; Y5 is direct seeding; B0 is submerged for 0 days (CK); B1 is submerged for 4 days; B2 is submerged for 7 days; B3 is submerged for 10 days. ± B0 represents the yield reduction rate of different treatments compared with the control of the non - submerged treatment. One-way analysis of variance (ANOVA) and the least significant difference (LSD) method were used for multiple comparisons. Different lowercase letters indicate significant differences among treatments (*P*< 0.05).

**Table 10:** Impact of submergence treatment on theoretical yield, including block theoretical yield (kg/hm^2^) and ± B0(%).

Treatment	Theoretical yield (kg/hm^2^)				± B0 (%)		
	B0	B1	B2	B3	B1	B2	B3
Y1	8869.22 ± 1014.75*a*	7643.70 ± 1519.91*a*	7398.62 ± 925.78*a*	5100.95 ± 919.96*b*	-13.82	-16.58	-42.49
Y2	10010.31 ± 757.73*a*	8953.65 ± 2936.18*a*	7763.15 ± 1499.19*a*	5674.54 ± 1574.67*ab*	-10.56	-22.45	-43.31
Y3	9146.38 ± 1672.61*a*	8073.93 ± 733.42*a*	7710.88 ± 1121.66*a*	6133.19 ± 2028.60*ab*	-11.73	-15.69	-32.94
Y4	10123.52 ± 1320.80*a*	9120.26 ± 2716.40*a*	8796.89 ± 337.25*a*	7099.91 ± 786.28*a*	-9.91	-13.1	-29.87
Y5	6747.66 ± 948.84*b*	4794.20 ± 1819.78*b*	4324.51 ± 1675.00*b*	3019.26 ± 703.00*c*	-28.95	-35.91	-55.25

Remark: Y1 is dry nursery seedlings + manual transplanting; Y2 is wet nursery seedlings + manual transplanting; Y3 is nutrient soil nursery seedlings + mechanical transplanting; Y4 is hard ground substrate micro - sprinkler nursery seedlings + mechanical transplanting; Y5 is direct seeding; B0 is submerged for 0 days (CK); B1 is submerged for 4 days; B2 is submerged for 7 days; B3 is submerged for 10 days. ± B0 represents the yield reduction rate of different treatments compared with the control of the non - submerged treatment. One-way analysis of variance (ANOVA) and the least significant difference (LSD) method were used for multiple comparisons. Different lowercase letters indicate significant differences among treatments (*P<* 0.05).

### 3.6 Impact of submergence treatment on submergence tolerance index and comprehensive evaluation of different rice cultivation methods

The submergence tolerance index is calculated as the ratio of the measured values under submergence conditions to those under normal water management conditions. This experiment systematically analyzed the submergence tolerance index for nineteen indicators, including tiller number, plant height, and number of green leaves, across five cultivation methods. An index greater than 1 indicates an increase, while an index less than 1 indicates a decrease.

As shown in **Supplementary Table S8**, after 7 days of submergence, there is an increasing trend in plant height, antioxidant enzyme activity, malondialdehyde (MDA), and proline content, indicating a pronounced response to submergence stress. Submergence significantly decreases tiller number and the number of green leaves within 7 days, while plant height increases. However, after more than 10 days of submergence, plant height starts to decrease due to the death of the main stem. The submergence tolerance index varies significantly among different seedling cultivation methods, indicating distinct differences in their responses to submergence stress.

Principal component analysis (PCA) was conducted on various indicators. These indicators included tiller number, plant height, SPAD value, POD activity, SOD activity, and MDA content. The contribution rates of the first four principal components were 58.52%, 19.29%, 16.43%, and 5.76%, respectively, and the cumulative contribution rate of these four principal components was 100%. This indicates that these four principal components retain most of the information from the original traits. The indicators that contributed the most to the first principal component (PC1) were MDA content, root - to - shoot ratio, tiller number, and number of green leaves. The indicators that contributed the most to the second principal component (PC2) were POD activity and SOD activity. The indicators that contributed the most to the third principal component (PC3) were PRO content, plant height, and SPAD value. Subordination analysis was conducted on the five cultivation methods, combining various evaluation indicators. The submergence tolerance performance of the five cultivation methods is as follows: Y4 > Y2 > Y1 > Y3 > Y5 (**Table 11**).

**Table 11:** Comprehensive evaluation of submergence tolerance of rice under different cultivation methods after 7 days of submergence.

Treatment	PC1	PC2	PC3	PC4	U(X1)	U(X2)	U(X3)	U(X4)	D value	Rank
Y4	0.78	3.39	0.14	-0.10	0.79	1.00	0.65	0.46	0.79	1
Y2	2.50	-1.25	1.04	-1.43	1.00	0.00	0.84	0.00	0.72	2
Y1	0.87	-0.88	1.78	1.45	0.80	0.08	1.00	1.00	0.71	3
Y3	1.68	-0.82	-2.87	0.39	0.00	0.09	0.00	0.63	0.58	4
Y5	-5.83	-0.45	-0.09	-0.30	0.00	0.17	0.60	0.39	0.15	5

Remark: Y1 is dry nursery seedlings + manual transplanting; Y2 is wet nursery seedlings + manual transplanting; Y3 is nutrient soil nursery seedlings + mechanical transplanting; Y4 is hard ground substrate micro-sprinkler nursery seedlings + mechanical transplanting; Y5 is direct seeding.

PCA was conducted on various indicators, with the contribution rates of the first three principal components being 45.13%, 40.22%, and 9.91%, respectively, accumulating to 95.27%. This indicates that these three principal components retain most of the information from the original traits. The indicators that contributed the most to the first principal component (PC1) were grains per panicle, effective panicles, plant height, root-to-shoot ratio, and seed setting rate. The indicators that contributed the most to the second principal component (PC2) were chlorophyll content, root-to-shoot ratio, thousand-grain weight, POD activity, and SOD activity. The average panicle length contributed the most to the third principal component (PC3).

The submergence tolerance index was calculated for nineteen indicators after 10 days of submergence for five cultivation methods, with the number of green leaves and SPAD value showing the most significant response to submergence stress. Compared to the other cultivation methods, the wet seedling cultivation and dry seedling cultivation methods exhibited a more pronounced increase in antioxidant enzyme activity (**Supplementary Table S8**). Subordination analysis was conducted on the five cultivation methods, combining various evaluation indicators. The submergence tolerance performance of the five cultivation methods is as follows: Y1 > Y2 > Y4 > Y3 > Y5 (**Table 12**).

**Table 12:** Comprehensive evaluation of submergence tolerance of rice under different cultivation methods after10 days of submergence.

Treatment	PC1	PC2	PC3	U(X1)	U(X2)	U(X3)	D value	Rank
Y1	1.48	3.59	-0.40	0.95	1.00	0.27	0.90	1
Y2	1.80	1.33	-0.06	1.00	0.69	0.37	0.80	2
Y4	0.32	-1.55	2.28	0.79	0.29	1.00	0.60	3
Y3	1.54	-3.67	-1.41	0.96	0.00	0.00	0.46	4
Y5	-5.14	-0.30	-0.42	0.00	0.55	0.27	0.26	5

Remark: Y1 is dry nursery seedlings + manual transplanting; Y2 is wet nursery seedlings + manual transplanting; Y3 is nutrient soil nursery seedlings + mechanical transplanting; Y4 is hard ground substrate micro-sprinkler nursery seedlings + mechanical transplanting; Y5 is direct seeding.

## 4 Comprehensive discussion

During the tillering stage, submergence leads to a series of changes in rice growth, development, and yield traits, catalyzing reactions that impact these parameters. As the duration of submergence increases, rice plant height decreases, heading stage is delayed, the number of effective panicles, grains per panicle, and thousand-grain weight are reduced. The longer the submergence period, the greater the impact, ultimately resulting in yield reduction (Xue et al., 2020; Hu et al., 2022; Kato et al., 2020). Submergence duration is a significant factor affecting rice yield reduction, with yield significantly decreasing as submergence duration increases. Additionally, submergence significantly delays the rice development stages (Islam et al., 2018). Research indicates that with increased submergence depth and duration, the tillering, booting, and heading stages of rice are delayed to varying degrees. Submergence promotes elongation growth of rice, affecting the rate of new tiller emergence and the survival of subsequent tillers, leading to increased plant height, reduced tiller number, and decreased yield (Ren et al., 2023).

Meanwhile, tiller growth is severely inhibited, plant height significantly increases, dry matter accumulation increases, and the number of effective panicles, seed setting rate, and actual yield significantly decrease (Wu et al., 2020). Our study shows that submergence stress leads to noticeable delays in growth stages. Within 7 days of submergence, plant height tends to increase, but beyond 10 days, plant height is significantly lower than the control. The impact of submergence stress on tiller number shows that within 4 days of submergence, tiller number does not differ much from the control; however, beyond 7 days, the tiller number significantly decreases compared to the control. This inconsistency with previous studies might be due to the difference in tillering ability between japonica and hybrid rice, requiring further experimental validation (Wang X. et al., 2021).

Moderate water stress can increase the content of antioxidant enzymes in rice plants. SOD and POD, as protective enzymes in the peroxide defense system, can defend against or mitigate damage to the cell membrane system by peroxidative free radicals, inhibiting membrane lipid peroxidation and reducing MDA content, thus alleviating stress - induced damage to plant cells (Das et al., 2022; Hao et al., 2013). In this study, the SOD activity in the control group was lower than in the submergence - treated group, indicating that submergence treatment is a relative stress condition, further proving that rice is a water - requiring but flood - intolerant plant. With increasing submergence duration, the POD activity, SOD activity, and free proline content in plant leaves significantly increased compared to the control, consistent with previous studies on submergence stress in various crops (Wang et al., 2022; Du et al., 2021; Thakur et al., 2024). Higher MDA content indicates a higher degree of membrane lipid peroxidation and more severe cell membrane damage. Our study found significant differences in leaf MDA content between the 7 - day and 10 - day submergence treatments compared to the control, with higher MDA content in the 10 - day submergence treatment. Proline (PRO) content in leaves increased significantly under all five seedling cultivation methods compared to the control, with differences among the cultivation methods. This indicates that under submergence stress during the tillering stage, rice leaves can maintain a high level of osmotic regulators with increasing submergence duration. The Y2 wet seedling cultivation and Y4 hard substrate seedling cultivation methods showed stronger osmotic regulation in rice leaves, reducing the damage caused by submergence stress (Chen et al., 2023).

Research indicates that the most severely and directly affected organs under flooding stress are the roots of plants (Wang et al., 2024). During submergence, as the oxygen supply around the plant roots is severely deficient, anaerobic respiration increases, which leads to the toxic effects of reactive oxygen species (ROS) and anaerobic metabolic by - products. With prolonged submergence, the toxic effects intensify, causing the roots to rot, turn black, and even die (Daniel and Hartman, 2024; Ugalde and Cardoso, 2023; Voesenek and Sasidharan, 2013). The root - to - shoot ratio, an important indicator reflecting the correlation between the underground and aboveground parts of plants, can be directly affected by flooding stress. Due to the damage and growth inhibition of rice roots caused by flooding, the fresh or dry weight of the underground part may decrease, affecting the root - to - shoot ratio (Anjum and Maiti, 2024). Simultaneously, the growth of the aboveground part may also be affected by reduced photosynthetic rates and nutrient absorption, further altering the root - to - shoot ratio (Wang et al., 2017). Our experimental results clearly show that the root - to - shoot ratio is relatively smallest under the direct seeding method, with the greatest impact under flooding stress. With prolonged flooding stress, the root - to - shoot ratio increases compared to the control. The primary reasons may be that the decline in the photosynthetic rate and nutrient accumulation in the above - ground part is greater than the damage to root growth, and that there is a substantial decrease in the dry weight of the above - ground part due to nutrient loss and the death of leaves and stems after submergence. However, the specific extent and mechanism of the impact may vary depending on rice variety, flooding severity, and flooding duration (Zhang et al., 2015; Barik et al., 2019; 55 Zhao et al., 2023). Therefore, a comprehensive understanding of the impact of flooding stress on the root - to - shoot ratio of rice requires further in - depth research and discussion.

Pot experiments investigating the impact of different submergence durations during various growth stages on rice growth and yield components have shown that submergence for up to 4 days during the tillering stage does not cause yield reduction compared to the control; submergence for 6 days results in an 80% yield reduction, essentially resulting in total yield loss; submergence for 8 and 10 days results in total plant death. Our experimental results differ significantly from these findings, with the direct seeding method being most sensitive to submergence during the tillering stage, showing noticeable yield reduction even with 4 days of submergence. In contrast, other seedling cultivation methods exhibit relatively smaller yield reductions, with a maximum yield reduction rate within 7 days of submergence. However, the maximum yield reduction rate after 10 days of submergence remains below 60%, and rice plants did not die completely. This experiment was rigorously designed in strict compliance with local rice cultivation practices, with the direct seeding operation specifically scheduled for the early days of June. The experimental outcomes unambiguously revealed that the direct seeding method manifested the highest degree of sensitivity to submergence stress during the tillering stage. A comprehensive analysis indicates that the most compelling rationale for this observation is the substantially delayed sowing date of direct seeding relative to the other four seedling - raising and transplanting methodologies. At the onset of the submergence treatment, the plant height of the directly - seeded rice was ascertained to be in the range of 40 to 50 centimeters, which was significantly lower compared to that of the transplanted rice. In conjunction with a comparatively smaller plant population density, this condition led to a diminished overall stress - resistance capability (Huang et al., 2016; Arai, 2022; Liang et al., 2023).

## 5 Conclusion

Within 7 days of submergence during the tillering stage of japonica rice in the Yangtze River basin and low-lying areas along the Huai River, a very good yield can still be achieved. Even if submergence lasts more than 10 days, a certain yield can still be obtained if reasonable remedial cultivation measures are taken and management is strengthened. Considering the yield reduction rate after submergence and comparing different seedling raising methods, regardless of whether the submergence duration is 7 days or 10 days, the direct seeding method has the worst submergence tolerance. Under the three seedling raising and transplanting modes of Y2, Y3 and Y4, the yield reduction rate within 7 days of flooding is all within 20%. However, when the flooding lasts for more than 10 days, the yield reduction rate increases significantly. Although the yield reduction rates of Y4 and Y3 are within 30%, the lowest yield reduction rate of Y4 also reaches 26.28%, and those of Y1, Y2 and Y5 are all above 40%. In the case of being submerged for 7 days, the comprehensive ranking of submergence tolerance is: Y4 > Y2 > Y1 > Y3 > Y5. When the submergence time reaches 10 days, the ranking is: Y1 > Y2 > Y4 > Y3 > Y5.

## Data Availability

The original contributions presented in the study are included in the article/**Supplementary Material**. Further inquiries can be directed to the corresponding author.
