# Supplementary material for: Differential adaptations of japonica rice to submergence stress during the tillering stage under various seedling cultivation and transplanting methods
Source: Front Plant Sci. 2025 Jun 19;16:1607055. doi: 10.3389/fpls.2025.1607055 (PMC12221938; doi:10.3389/fpls.2025.1607055)
Supplement: Supplementary file 1 [file SupplementaryFile1.docx]

**Supporting Information**

Differential Adaptations of Japonica Rice to Submergence Stress during the Tillering Stage under Various Seedling Cultivation and Transplanting Methods

Sumei Duan ^1,†,*^, Qianxi Zhang ^1,†^, Hao Ai ^1^, Tingting Feng ^1^, Aifeng Zhou ^2^, Yi Liu ^3^, Yuqin Wang ^3^, Fei Fang ^3^

1 Center for Crop Biotechnology, College of Agriculture, Anhui Science and Technology University, Chuzhou 239000, China; duansm@ahstu.edu.cn (S.D.); yjs2022253@163.com (Q.Z.); aihao@ahstu.edu.cn (H.A.); fengtingtingyt@ahstu.edu.cn (T.F.)

2 Anhui Xin Fu Xiang Tian Ecological Agriculture Co. Ltd., Ma’anshan 238200, China; xinfuxiangtian@126.com (A.Z.)

3 Ma’anshan Agriculture and Rural Bureau, Ma’anshan, 243011, China; maszzglz@126.com (Y.L.); wh2008sh@163.com (Y.W.); maszbz@163.com (F.F.)

^†^ These authors contributed equally to this work.

*Correspondence: duansm@ahstu.edu.cn

**Table S1**. Changes in agronomic traits across treatments (submergence duration: 4 days).

| **Seedling method** | **Relative change rate of plant height** | **Relative survival rate of green leaves** | **Tiller mortality rate** | **Tiller growth rate** |
| --- | --- | --- | --- | --- |
| **Y1** | 1.03±0.07*abc* | 0.86±0.12*a* | 0.14±0.19*bcd* | 0.11±*ab* |
| **Y2** | 0.97±0.06*bcd* | 0.75±0.12*abcd* | 0.19±0.12*bcd* | -0.19±*bc* |
| **Y3** | 0.97±0.05*bcd* | 0.84±0.1*ab* | 0.39±0.35*abc* | -0.38±*bc* |
| **Y4** | 0.92±0.09*bcd* | 0.8±0.22a*bc* | 0.00±0.11*d* | 0.3±*ab* |
| **Y5** | 0.99±0.13*bcd* | 0.59±0.06*cde* | 0.49±0.04*ab* | 0±*ab* |

Remark: Y1 is dry nursery seedlings + manual transplanting, Y2 is wet nursery seedlings + manual transplanting, Y3 is nutrient soil nursery seedlings + mechanical transplanting, Y4 is hard ground substrate micro - sprinkler nursery seedlings + mechanical transplanting, Y5 is direct seeding; One-way analysis of variance (ANOVA) and the least significant difference (LSD) method were used for multiple comparisons. Different lowercase letters indicate significant differences among treatments (*P* < 0.05).

**Table S2**. Changes in agronomic traits across treatments (submergence duration: 7 days).

| **Seedling method** | **Relative change rate of plant height** | **Relative survival rate of green leaves** | **Tiller mortality rate** | **Tiller growth rate** |
| --- | --- | --- | --- | --- |
| **Y1** | 1.12±0.07*ab* | 0.70±0.17*abcde* | 0.25±0.17*abcd* | -0.75±0.05*bcd* |
| **Y2** | 0.96±0.09*bcd* | 0.50±0.18*ef* | 0.36±0.06*abc* | -0.27±0.03*bc* |
| **Y3** | 1.06±0.06*abc* | 0.54±0.12*def* | 0.59±0.08*a* | -1.2±0.02*cd* |
| **Y4** | 0.92±0.14*cd* | 0.53±0.05*def* | 0.11±0.1*cd* | 0.98±0.01*a* |
| **Y5** | 1.20±0.12*a* | 0.33±0.08*fgh* | 0.39±0.35*abc* | -0.42*b*±0.02*c* |

Remark: Y1 is dry nursery seedlings + manual transplanting, Y2 is wet nursery seedlings + manual transplanting, Y3 is nutrient soil nursery seedlings + mechanical transplanting, Y4 is hard ground substrate micro - sprinkler nursery seedlings + mechanical transplanting, Y5 is direct seeding; One-way analysis of variance (ANOVA) and the least significant difference (LSD) method were used for multiple comparisons. Different lowercase letters indicate significant differences among treatments (*P* < 0.05).

**Table S3**. Changes in agronomic traits across treatments (submergence duration: 10 days).

| **Seedling method** | **Relative change rate of plant height** | **Relative survival rate of green leaves** | **Tiller mortality rate** | **Tiller growth rate** |
| --- | --- | --- | --- | --- |
| **Y1** | 1.02±0.14*abc* | 0.61±0.14*bcde* | 0.28±0.08*abcd* | -0.34±0.03*bc* |
| **Y2** | 0.95±0.09*bcd* | 0.47±0.16*efg* | 0.61±0.07*a* | -0.13±0.01*abc* |
| **Y3** | 0.87±0.06*cd* | 0.12±0.07*h* | 0.27±0.13*abcd* | -1.85±0.02*d* |
| **Y4** | 0.81±0.1*d* | 0.25±0.11*gh* | 0.26±0.21*abcd* | -0.08±0.03*abc* |
| **Y5** | 1.00±0.11*bcd* | 0.24±0.11*h* | 0.61±0.25*a* | -1.22±0.02*cd* |

Remark: Y1 is dry nursery seedlings + manual transplanting, Y2 is wet nursery seedlings + manual transplanting, Y3 is nutrient soil nursery seedlings + mechanical transplanting, Y4 is hard ground substrate micro - sprinkler nursery seedlings + mechanical transplanting, Y5 is direct seeding; One-way analysis of variance (ANOVA) and the least significant difference (LSD) method were used for multiple comparisons. Different lowercase letters indicate significant differences among treatments (*P* < 0.05).

**Table S4**. Effects of different treatments on yield components.

| **reatment/Item** | **Effective Panicles (number/hill)** | **Panicle Length (cm)** | **Grains per Panicle (number/hill)** | **Aboveground Biomass (g/hill)** | **Harvest Index** |
| --- | --- | --- | --- | --- | --- |
| **Y1B0** | 15.00±3.00*abc* | 12.38±0.49*cd* | 1369.33±151.35*abcd* | 58.41±5.68*cdef* | 0.53±0.01*bcd* |
| **Y1B1** | 12.67±4.51*cde* | 13.75±1.08*bcd* | 1766.00±573.66*a* | 67.83±3.95*bcde* | 0.47±0.12*cdef* |
| **Y1B2** | 12.00±3.00*cde* | 14.23±0.84*abcd* | 1220.67±37.87*bcde* | 56.73±13.23*def* | 0.44±0.02*cdef* |
| **Y1B3** | 8.33±2.08*fg* | 14.43±3.41*abcd* | 924.67±86.52*efg* | 53.02±12.77*defg* | 0.46±0.08*cdef* |
| **Y2B0** | 18.00±6.00*a* | 14.19±0.95*abcd* | 1787.00±225.86*a* | 91.53±20.08*a* | 0.41±0.04*cf* |
| **Y2B1** | 13.00±5.57*cd* | 12.79±1.84*bcd* | 1434.33±458.87*abcd* | 66.36±37.08*bcde* | 0.50±0.03*cdef* |
| **Y2B2** | 12.00±5.2*cde* | 11.73±4.94*cd* | 1267.33±234.15*bcde* | 79.52±6.76*ab* | 0.42±0.02*def* |
| **Y2B3** | 13.67±2.08*bcd* | 12.2±0.36*cd* | 991.67±222.79*defg* | 40.77±9.02*fghi* | 0.54±0.01*bc* |
| **Y3B0** | 10.67±2.08*def* | 11.18±4.5*cd* | 1296.67±201.14*bcde* | 72.52±8.29*bcd* | 0.47±0.02*cdef* |
| **Y3B1** | 11.67±0.58*cdef* | 12.85±3.1*bcd* | 916.67±168.1*efg* | 49.01±*efgh* | 0.46±0.07*cdef* |
| **Y3B2** | 14.33±1.53*bcd* | 15.01±1.08*abc* | 1129±94.43*cdef* | 58.46±5.46*cdef* | 0.52±0.02*bcde* |
| **Y3B3** | 11.00±2.65*def* | 17.15±6.67*ab* | 1000.33±165.17*defg* | 34.24±5.55*ghi* | 0.66±0.04*a* |
| **Y4B0** | 16.67±1.53*ab* | 10.03±5.64*d* | 1600.33±182.62*ab* | 77.56±9.06*abc* | 0.53±0.02*bcd* |
| **Y4B1** | 13.33±1.53*bcd* | 14.34±1.18*abcd* | 1519.67±496.48*abc* | 68.13±*bcde* | 0.49±0.19*cdef* |
| **Y4B2** | 9.33±1.53*efg* | 10.29±4.11*d* | 1560.00±34.66*abc* | 52.86±21.34*defg* | 0.46±0.03*cdef* |
| **Y4B3** | 8.33±1.53*fg* | 10.63±4.31*cd* | 1287.33±66.11*bcde* | 31.27±6.55*hi* | 0.60±0.07*cdef* |
| **Y5B0** | 7.00±1.00*gh* | 13.67±1.15*bcd* | 1029.33±121.06*defg* | 43.56±2.24*fgh* | 0.42±0.03*def* |
| **Y5B1** | 4.33±3.21*hi* | 13.38±0.53*bcd* | 763.00±212.45*fg* | 29.87±0.35*hi* | 0.50±0.01*bcdef* |
| **Y5B2** | 6.00±1.00*gh* | 18.26±0.35*a* | 777.67±199.61*fg* | 31.73±2.74*hi* | 0.40±0.03*f* |
| **Y5B3** | 2.00±0.00*i* | 14.2±1.02*abcd* | 600.67±82.92*g* | 23.30±3.58*i* | 0.39±0.07*f* |

Remark: Y1 is dry nursery seedlings + manual transplanting, Y2 is wet nursery seedlings + manual transplanting, Y3 is nutrient soil nursery seedlings + mechanical transplanting, Y4 is hard ground substrate micro - sprinkler nursery seedlings + mechanical transplanting, Y5 is direct seeding; B0 is submerged for 0 days (CK), B1 is submerged for 4 days, B2 is submerged for 7 days, B3 is submerged for 10 days; One-way analysis of variance (ANOVA) and the least significant difference (LSD) method were used for multiple comparisons. Different lowercase letters indicate significant differences among treatments (*P* < 0.05).

**Table S5**. F-values for Yield Components.

| **reatment/**  **Item** | **Effective Panicles (number/hill)** | **Panicle Length (cm)** | **Grains per Panicle (number/hill)** | **Aboveground Biomass (g/hill)** | **Harvest Index** | **degree of freedom（DF)** |
| --- | --- | --- | --- | --- | --- | --- |
| **between blocks** | 29.753** | 19.22** | 5.60** | 12.10** | 3.48* | 2 |
| **between treatments** | 13.662** | 2.67** | 6.50** | 10.14** | 3.92** | 19 |
| **Cultivation (Y)** | 41.999** | 4.49** | 17.53** | 21.50** | 5.80** | 4 |
| **Submergence Duration (B)** | 13.301** | 1.59 | 10.47** | 25.09** | 4.92** | 3 |
| **Y × B** | 4.306** | 2.33* | 1.83 | 2.62* | 3.04 | 12 |

**Table S6**. Effects of different treatments on yield components.

| **Treatment/Item** | **Filled Grains (number/hill)** | **Shriveled Grains (number/hill)** | **Seed Setting Rate (%)** | **Thousand-Grain Weight (g)** | **Theoretical Yield (kg/hm^2^)** |
| --- | --- | --- | --- | --- | --- |
| **Y1B0** | **1195.33±116.32*bcde*** | **174.00±46.12*b*** | **87.39±2.22*ab*** | **26.67±0.49*bcde*** | **8869.22±101.75*ab*** |
| **Y1B1** | **1046.67±185.23*cdefg*** | **617.67±96.37*a*** | **71.8±28.95*cd*** | **26.21±0.95*cdef*** | **7643.70±159.91*bcde*** |
| **Y1B2** | **1013.33±68.97*defg*** | **207.33±33.08*b*** | **82.95±3.25*abc*** | **26.23±2.09*cdef*** | **7398.62±925.78*bcde*** |
| **Y1B3** | **660.67±71.30*ij*** | **264.00±18.19*b*** | **71.38±1.45*cd*** | **27.63±2.07*abc*** | **5100.95±919.96*gh*** |
| **Y2B0** | **1453.33±73.24*a*** | **333.67±153.97*ab*** | **81.82±5.91*abc*** | **24.76±0.62*ef*** | **10010.31±757.73*a*** |
| **Y2B1** | **1236.67±380.01*abcd*** | **197.67±93.95*b*** | **86.68±4.00*ab*** | **25.91±0.9*cdef*** | **8953.65±2936.18*ab*** |
| **Y2B2** | **1083.67±174.67cdef** | **183.67±69.62b** | **85.75±3.24*abc*** | **25.70±0.94*cdef*** | **7763.15±1499.19*bcde*** |
| **Y2B3** | **763.00±180.55*hij*** | **228.67±45.46*b*** | **76.78±2.03*bcd*** | **26.59±1.12*bcde*** | **5674.54±157.67*fgh*** |
| **Y3B0** | **1192.33±193.06*bcde*** | **104.33±8.08*b*** | **91.89±0.63*a*** | **27.54±0.57*abcd*** | **9146.38±167.61*ab*** |
| **Y3B1** | **1081.67±152.01*cdef*** | **81.67±35.52*b*** | **91.07±3.25*ab*** | **27.00±1.59*abcd*** | **8073.93±733.42*bcd*** |
| **Y3B2** | **954.33±101.28*efgh*** | **174.67±28.57*b*** | **84.44±2.89*abc*** | **28.99±1.24*a*** | **7710.88±1121.66*bcde*** |
| **Y3B3** | **825.67±206.29*ghi*** | **174.67±51.96*b*** | **81.79±7.35*abc*** | **26.36±2.21*bcde*** | **6133.19±2028.60*efg*** |
| **Y4B0** | **1427.33±154.46*ab*** | **173.00±31.00*b*** | **89.24±1.00*ab*** | **25.48±0.60*def*** | **10123.52±1320.80*a*** |
| **Y4B1** | **1281.00±368.15*abc*** | **238.67±128.52*b*** | **85.14±4.04*abc*** | **25.54±1.35*cdef*** | **9120.26±2716.04*ab*** |
| **Y4B2** | **1281.33±50.62*abc*** | **278.67±20.26*b*** | **82.12±1.60*abc*** | **24.72±1.08*ef*** | **8796.89±337.25*abc*** |
| **Y4B3** | **1037.67±28.54*cdefg*** | **249.67±44.74*b*** | **80.69±2.61*abc*** | **24.65±3.03*ef*** | **7099.91±786.28*cdef*** |
| **Y5B0** | **929.00±111.50*fgh*** | **100.33±22.19*b*** | **90.24±1.79*ab*** | **26.11±1.30*cdef*** | **6747.66±948.84*def*** |
| **Y5B1** | **700.33±216.23*ij*** | **62.67±15.31*b*** | **91.13±4.14*ab*** | **24.17±2.31*f*** | **4794.20±1819.78*gh*** |
| **Y5B2** | **563.67±190.80*jk*** | **214.00±280.35*b*** | **71.63±6.15*cd*** | **27.33±1.32*abcd*** | **4324.51±167.00*hi*** |
| **Y5B3** | **382.33±84.05*k*** | **218.33±49.37*b*** | **63.36±7.99*d*** | **28.39±0.94*ab*** | **3019.26±703.00*i*** |

Remark: Y1 is dry nursery seedlings + manual transplanting, Y2 is wet nursery seedlings + manual transplanting, Y3 is nutrient soil nursery seedlings + mechanical transplanting, Y4 is hard ground substrate micro - sprinkler nursery seedlings + mechanical transplanting, Y5 is direct seeding; B0 is submerged for 0 days (CK), B1 is submerged for 4 days, B2 is submerged for 7 days, B3 is submerged for 10 days; One-way analysis of variance (ANOVA) and the least significant difference (LSD) method were used for multiple comparisons. Different lowercase letters indicate significant differences among treatments (*P* < 0.05).

**Table S7**. F-values for Yield Components.

| **Treatment/**  **Item** | **Filled Grains (number/hill)** | **Shriveled Grains (number/hill)** | **Seed Setting Rate (%)** | **Thousand-Grain Weight (g)** | **Theoretical Yield (kg/hm^2^)** | **degree of freedom（DF)** |
| --- | --- | --- | --- | --- | --- | --- |
| **between blocks** | 21.178** | 0.357 | 1.435 | 19.11** | 35.26** | 2 |
| **between treatments** | 15.298** | 1.149 | 3.282** | 4.13** | 13.93** | 19 |
| **Cultivation (Y)** | 39.248** | 1.849 | 2.936* | 8.52** | 35.10** | 4 |
| **Submergence Duration (B)** | 41.261** | 0.309 | 8.853** | 2.51 | 39.70** | 3 |
| **Y × B** | 0.824 | 1.125 | 2.004 | 3.08** | 0.43 | 12 |

**Table S8**. Submergence tolerance index of rice under different cultivation methods at 7 days and 10 days.

| **Treatment/Item** | **B2** | | | | | **B3** | | | | |
| --- | --- | --- | --- | --- | --- | --- | --- | --- | --- | --- |
|  | **Y1** | **Y2** | **Y3** | **Y4** | **Y5** | **Y1** | **Y2** | **Y3** | **Y4** | **Y5** |
| **Tiller number** | 0.94 | 0.95 | 0.72 | 0.67 | 0.43 | 0.82 | 0.76 | 0.47 | 0.51 | 0.44 |
| **Plant height** | 1.09 | 1.02 | 1.02 | 0.95 | 1.19 | 0.97 | 0.80 | 0.77 | 0.81 | 0.90 |
| **Number of green leaves** | 0.87 | 0.34 | 0.41 | 1.06 | 0.46 | 0.60 | 0.45 | 0.13 | 0.22 | 0.23 |
| **SPAD value** | 1.02 | 1.13 | 0.94 | 0.93 | 0.96 | 0.96 | 0.93 | 0.70 | 0.82 | 0.76 |
| **Root-to-shoot ratio** | 1.47 | 1.88 | 1.58 | 2.11 | 2.67 | 0.95 | 1.06 | 1.63 | 2.21 | 1.83 |
| **POD** | 1.13 | 1.57 | 1.06 | 1.08 | 1.12 | 1.17 | 1.91 | 1.10 | 1.06 | 1.11 |
| **SOD** | 1.12 | 1.57 | 1.21 | 1.12 | 1.07 | 1.29 | 1.83 | 1.15 | 1.15 | 1.13 |
| **MDA** | 1.97 | 1.34 | 1.81 | 1.66 | 1.26 | 2.83 | 2.19 | 1.67 | 2.18 | 2.15 |
| **PRO** | 1.30 | 1.47 | 1.21 | 1.17 | 1.70 | 1.33 | 1.07 | 1.54 | 1.77 | 1.80 |
| **Effective number of spikes** | 0.67 | 0.56 | 1.34 | 0.56 | 0.86 | 0.76 | 0.80 | 1.03 | 0.50 | 0.29 |
| **Average spike length** | 0.83 | 1.15 | 1.34 | 1.03 | 1.34 | 0.86 | 1.17 | 1.53 | 1.06 | 1.04 |
| **Number of grains per ear** | 0.71 | 0.89 | 0.87 | 0.97 | 0.76 | 0.55 | 0.68 | 0.77 | 0.80 | 0.58 |
| **Biomass** | 0.87 | 0.97 | 0.81 | 0.68 | 0.73 | 0.45 | 0.91 | 0.47 | 0.40 | 0.53 |
| **Economic coefficient** | 1.02 | 0.83 | 1.10 | 0.87 | 0.96 | 1.32 | 0.87 | 1.39 | 1.13 | 0.93 |
| **Actual number of particles** | 0.75 | 0.85 | 0.80 | 0.90 | 0.61 | 0.53 | 0.55 | 0.69 | 0.73 | 0.41 |
| **Solidity rate** | 1.05 | 0.95 | 0.92 | 0.92 | 0.79 | 0.94 | 0.82 | 0.89 | 0.90 | 0.70 |
| **1000 - grain weight** | 1.04 | 0.98 | 1.05 | 0.97 | 1.05 | 1.07 | 1.04 | 0.96 | 0.97 | 1.09 |
| **Theoretical yield** | 0.78 | 0.83 | 0.84 | 0.87 | 0.64 | 0.57 | 0.58 | 0.67 | 0.70 | 0.45 |
| **Actual output** | 0.81 | 0.76 | 0.82 | 0.87 | 0.65 | 0.59 | 0.57 | 0.70 | 0.74 | 0.43 |

Remark: Y1 is dry nursery seedlings + manual transplanting, Y2 is wet nursery seedlings + manual transplanting, Y3 is nutrient soil nursery seedlings + mechanical transplanting, Y4 is hard ground substrate micro - sprinkler nursery seedlings + mechanical transplanting, Y5 is direct seeding; B0 is submerged for 0 days (CK), B2 is submerged for 7 days, B3 is submerged for 10 days; Different lowercase letters in the same column indicate significant differences (*P* < 0.05).

**Supplementary Material 9: Technical Key Points of Different Seedling - raising Methods for Y1 - Y4**

**1. Y1 Dry - nursery and Manual Transplanting of Rice Seedlings:**

**I. Seedbed Preparation**

**Site Selection**

Choose dry land or paddy fields with high terrain, good drainage, fertile soil, loose and breathable texture, close to water sources, and free from diseases, pests, and weeds as the seedbed. It is preferably slightly acidic soil, with a pH value of 4.5 - 5.5 being appropriate.

**Soil Fertilization**

15 - 20 days before sowing, apply 10 - 15 kg of fully decomposed farmyard manure, 0.2 - 0.3 kg of superphosphate, and 0.1 - 0.15 kg of potassium sulfate per square meter of the seedbed. Then plow the soil to thoroughly mix the fertilizers with the soil.

**Seedbed Making**

The seedbed is generally made into a high - ridge bed with a width of 1.2 - 1.5 m and a height of 0.15 - 0.2 m. The length is determined according to the terrain and planting area. The bed surface should be flat and finely broken, and drainage ditches should be dug around it to facilitate drainage.

**II. Seed Treatment**

**Sun - drying Seeds**

2 - 3 days before sowing, spread the seeds on bamboo mats or cement floors and sun - dry them for 1 - 2 days. Turn the seeds 3 - 4 times a day to improve the germination potential and germination rate.

**Seed Selection**

Select seeds with clean water or saltwater to remove empty grains, impurities, and diseased grains. When using saltwater for seed selection, the appropriate concentration of saltwater is 1.05 - 1.10 g/cm³. After seed selection, rinse the seeds with clean water.

**Soaking and Disinfecting Seeds**

Soak the selected seeds in a 2000 - 3000 - fold solution of 25% prochloraz EC or other suitable agents for 36 - 48 hours to prevent diseases such as bakanae disease. After soaking, rinse the seeds thoroughly with clean water.

**Germination Promotion**

Wrap the soaked seeds with a damp cloth and place them in an environment of 30 - 32°C for germination. When the rate of seeds showing white after breaking the hull reaches over 80%, lower the temperature to about 25°C, spread out the seeds to air - dry the buds for 6 - 8 hours, and then they are ready for sowing.

**III. Sowing**

**Determining the Sowing Date**

Determine the appropriate sowing date according to local climate conditions and the characteristics of the rice variety. Generally, it is advisable to start sowing when the local daily average temperature stably exceeds 10°C.

**Controlling the Sowing Rate**

Determine the sowing rate according to the seedling age and variety characteristics. Generally, the sowing rate for conventional rice is 25 - 30 kg per mu of the seedbed, and for hybrid rice, it is 10 - 15 kg per mu of the seedbed.

**Sowing Method**

Evenly spread the seeds on the bed surface, then gently press them with a wooden board so that the seeds are embedded in the soil on three sides. Cover the seeds with sieved fine soil, with a thickness of 0.5 - 1 cm. After covering, spray a special herbicide for dry - nursery rice seedlings on the bed surface, and then cover with plastic film or build a small arched shed to keep warm and moist.

**IV. Seedbed Management**

**Temperature Management**

From sowing to emergence, focus on maintaining warmth and moisture, and control the temperature inside the film at 30 - 35°C. From emergence to the one - leaf - one - heart stage, control the temperature at 25 - 30°C. From the one - leaf - one - heart stage to the two - leaf - one - heart stage, control the temperature at 20 - 25°C. After the two - leaf - one - heart stage, gradually uncover the film for hardening the seedlings. In case of low temperatures, cover the film in time to keep warm.

**Water Management**

For dry - nursery rice seedlings, adhere to the principle of "dry rather than wet". Keep the bed soil moist before emergence to facilitate seedling emergence. After emergence, water as little as possible to promote root penetration. When the seedlings show water - shortage symptoms such as rolled leaves and no water exudation at dawn and dusk, water thoroughly in time.

**Fertilization Management**

At the two - leaf - one - heart stage, spray 5 - 7 kg of urea and 3 - 5 kg of potassium sulfate per mu of the seedbed, diluted with 1000 - 1500 kg of water for foliar application. After application, rinse the leaves with clean water to prevent leaf - burning. Subsequently, top - dress fertilizers as appropriate according to the growth of the seedlings.

**Pest and Disease Control**

Focus on preventing and controlling diseases and pests such as damping - off, bacterial wilt, and rice thrips. At the one - leaf - one - heart stage, spray a 1000 - 1500 - fold solution of 30% hymexazol + metalaxyl aqueous solution per mu of the seedbed to prevent damping - off. Use imidacloprid and other agents to control pests such as rice thrips.

**V. Transplanting**

**Seedling Uprooting**

Water the seedbed thoroughly 1 - 2 days before uprooting the seedlings to facilitate uprooting. When uprooting the seedlings, try to damage the roots as little as possible, keep as much soil as possible around the roots, and maintain the integrity of the root system.

**Transplanting Method**

Both manual transplanting and mechanical transplanting can be used. During transplanting, ensure shallow and even planting. The transplanting depth should be about 2 - 3 cm. Determine the plant spacing and row spacing reasonably according to the variety and planting density requirements.

**2. Y2 Wet Nursery and Manual Transplanting of Rice Seedlings: This is a common method of manual transplanting for rice seedling cultivation, and the key technical points are as follows.**

**I. Seedling - bed Preparation**

**Site Selection and Land Preparation**

Select fields with flat terrain, convenient irrigation and drainage, fertile soil, and few weeds as the seedling - bed. Conduct fine land preparation before sowing to make the field surface flat, with a height difference of no more than 3 cm. The soil should be soft on the top and loose at the bottom, with a moderate texture.

**Applying Sufficient Base Fertilizer**

Generally, before plowing, apply 15 - 22.5 tons of decomposed farmyard manure per hectare, or 300 - 450 kg of special compound fertilizer for rice. Then plow the fertilizer evenly into the soil to fully mix the soil and fertilizer, providing sufficient nutrients for the growth of rice seedlings.

**Making Ridges and Ditches**

Make the seedling - bed into ridges with a width of 1.5 - 1.8 m. The ridge ditches should be 30 - 40 cm wide and 15 - 20 cm deep, and surround the field with ditches for good drainage and management.

**II. Seed Treatment**

**Sun - drying Seeds**

2 - 3 days before sowing, on sunny days, spread the seeds on bamboo trays or cement floors. The thickness is preferably 2 - 3 cm. Turn the seeds 3 - 4 times a day, and the sun - drying time is 2 - 3 hours to enhance seed vitality and germination potential.

**Seed Selection**

Use saltwater or muddy water for seed selection. The specific gravity of saltwater or muddy water is generally 1.05 - 1.10. Scoop out the empty grains and impurities floating on the surface, and select plump seeds. After selection, rinse the seeds with clean water.

**Soaking and Disinfecting Seeds**

Soak the seeds in clean water to make them fully absorb water. The general soaking time is 3 - 5 days, and change the water 1 - 2 times a day during this period. At the same time, use agents such as strong chlorine - containing disinfectant to disinfect the seeds to prevent diseases such as bakanae disease.

**Germination Promotion**

Wrap the soaked seeds with a damp cloth or sack and place them in an environment of 30 - 32°C for germination. When the rate of seeds showing white after breaking the hull reaches over 80%, lower the temperature to about 25°C and conduct bud - hardening for 12 - 24 hours. When the bud length is half the length of a grain of rice and the root length is the length of one grain of rice, the seeds are ready for sowing.

**III. Sowing**

**Determining the Sowing Date**

Determine the appropriate sowing date according to local climate conditions, variety characteristics, and cropping arrangements. Generally, start sowing when the daily average temperature stably exceeds 12°C.

**Controlling the Sowing Rate**

The sowing rate for conventional rice is 150 - 200 g per square meter of the seedling - bed, and for hybrid rice, it is 100 - 120 g per square meter of the seedling - bed.

**Even Sowing**

Evenly sow the seeds on the seedling - bed ridges. To ensure uniform sowing, the method of sowing a fixed amount of seeds per ridge can be adopted. After sowing, gently press the seeds with a wooden board so that the seeds are half - buried in the soil. Then cover the seeds with sieved fine soil or plant ash, with a thickness of 0.5 - 1 cm.

**IV. Seedling - bed Management**

**Water Management**

From sowing to emergence, keep the seedling - bed moist to promote seed germination and root growth. From emergence to the two - leaf - one - heart stage, adopt the method of shallow and frequent irrigation, keeping the water layer depth in the seedling - bed at 1 - 2 cm. After the two - leaf - one - heart stage, gradually deepen the water layer to 3 - 5 cm to promote tillering of rice seedlings.

**Fertilization Management**

At the one - leaf - one - heart stage of the seedlings, apply 75 - 105 kg of urea per hectare as the "weaning fertilizer". 3 - 5 days before transplanting, apply 45 - 60 kg of urea per hectare as the "transplanting - promoting fertilizer" to enhance the stress resistance of the seedlings and their green - returning ability after transplanting.

**Pest and Disease Control**

The relatively high humidity in the wet - nursery environment makes it prone to pests and diseases. Focus on preventing and controlling diseases and pests such as damping - off, cottony rot, rice thrips, and stem borers. Regularly spray agents such as dexon to prevent damping - off, and use imidacloprid to control pests such as rice thrips.

**Weed Control**

2 - 3 days after sowing, spray 1500 - 2250 ml of 30% pretilachlor EC per hectare, diluted with 450 - 600 kg of water, to seal the weeds. After the three - leaf stage of the seedlings, for weeds such as barnyard grass, select agents such as penoxsulam for foliar treatment.

**V. Transplanting**

**Seedling Uprooting**

One day before uprooting the seedlings, drain the water in the seedling - bed to make the soil dry and easy for uprooting. When uprooting the seedlings, try to minimize damage to the root system of the seedlings, uproot the seedlings with as much soil as possible, and maintain the integrity of the root system.

**Transplanting Specifications**

Determine the transplanting specifications reasonably according to variety characteristics and planting density requirements. Generally, for hybrid rice, the row spacing is 25 - 30 cm and the plant spacing is 15 - 20 cm; for conventional rice, the row spacing is 20 - 25 cm and the plant spacing is 13 - 17 cm.

**3. Y3 Nutrient - soil Nursery and Machine - transplanting of Rice Seedlings: This is a common and effective method for raising rice seedlings. The following are its key technical points.**

**I. Nutrient - soil Preparation**

**Soil Selection**

Select garden soil, paddy - field soil, or dry - land soil that is fertile, loose, well - ventilated, and free from weed seeds and pathogens. Avoid using soil that is overly sandy or overly sticky.

**Fertilization and Amendment**

Generally, for every 1000 kg of nutrient - soil, 100 - 150 kg of fully decomposed farmyard manure, 5 - 10 kg of superphosphate, and 1 - 2 kg of potassium sulfate can be added. An appropriate amount of strong - seedling agent can also be added to enhance the quality of the seedlings. Then, thoroughly mix the fertilizers with the soil.

**Disinfection Treatment**

To prevent pests and diseases, the nutrient - soil needs to be disinfected. 70% dexon wettable powder can be used. Use 2 - 3 g per square meter of the seedbed, dilute it with 2 - 3 kg of water, spray it on the nutrient - soil, and then cover it with plastic film and pile it up for 2 - 3 days.

**II. Seedbed Preparation**

**Seedbed Selection**

Choose a plot with flat terrain, facing the sun and sheltered from the wind, convenient for irrigation and drainage, and close to the main field as the seedbed.

**Seedbed Making**

The seedbed is generally made into a high - ridge bed with a width of 1.2 - 1.5 m. The ridge ditch is 30 - 40 cm wide and 15 - 20 cm deep to facilitate drainage and management. The length of the seedbed is determined according to the actual situation, generally not exceeding 20 m for easy operation and management. Before sowing, rake and level the seedbed soil to make the bed surface flat and soft.

**III. Seed Treatment**

**Sun - drying Seeds**

Select sunny days before sowing and sun - dry the seeds for 1 - 2 days to improve the germination potential and germination rate of the seeds.

**Seed Selection**

Select seeds with clean water or saltwater to remove empty grains, impurities, and diseased grains, and retain plump and full seeds. When using saltwater for seed selection, the concentration of saltwater is generally 1.05 - 1.10 kg/L. After seed selection, rinse the seeds with clean water.

**Soaking and Disinfecting Seeds**

Soak the selected seeds in clean water to make them fully absorb water. At the same time, an appropriate amount of fungicides such as hymexazol and prochloraz can be added to the soaking water for seed disinfection to prevent diseases such as bakanae disease. The soaking time is generally 2 - 3 days, and it can be adjusted appropriately according to the water temperature.

**Germination Promotion**

After soaking, germinate the seeds at a temperature of 30 - 32°C. When the seeds show white after breaking the hull, lower the temperature to about 25°C and continue germinating until the bud length is 2 - 3 mm, then they are ready for sowing.

**IV. Sowing**

**Sowing Time**

Select an appropriate sowing time according to local climate conditions and the characteristics of the rice variety. Generally, start sowing when the temperature is stably above 10°C.

**Sowing Rate**

Determine the sowing rate according to factors such as the variety, seedling age, and seed - raising method. Generally, the sowing rate for conventional rice is 25 - 30 kg per mu of the seedbed, and for hybrid rice, it is 10 - 15 kg per mu of the seedbed.

**Sowing Method**

Evenly broadcast the germinated seeds on the well - watered seedbed, and then cover them with 0.5 - 1 cm thick nutrient - soil so that the seeds are not visible. The covering soil should be fine and even, and then gently press to make the seeds closely combine with the soil.

**V. Seedbed Management**

**Water Management**

From sowing to emergence, keep the seedbed moist but without waterlogging. From emergence to the 3 - leaf stage, mainly maintain a moist state. Keep the ditch full of water on sunny days, half - full on cloudy days, and drain the water on rainy days. After the 3 - leaf stage, appropriately control the water to promote the growth of the seedling roots.

**Temperature Management**

Before emergence after sowing, focus on heat preservation, and measures such as covering with plastic film can be adopted. When the temperature inside the film exceeds 35°C, ventilate in time to cool down. After emergence, uncover the film and ventilate for hardening the seedlings in a timely manner according to the weather conditions to prevent high - temperature damage to the seedlings.

**Fertilization Management**

At the 1 - leaf - 1 - heart stage and 3 - leaf - 1 - heart stage of the seedlings, top - dress a "weaning fertilizer" and a "relay fertilizer" respectively. Generally, use 5 - 7 kg of urea per mu, dilute it with 1000 - 1500 kg of water and spray it evenly. 3 - 5 days before transplanting, apply a "transplanting - promoting fertilizer", using 7 - 10 kg of urea per mu.

**Pest and Disease Control**

The main pests and diseases during the seedbed period include damping - off, bacterial wilt, rice thrips, and stem borers. Regularly check the growth of the seedlings, promptly detect pests and diseases, and take corresponding control measures. For example, damping - off can be controlled with dexon and other agents, and rice thrips can be controlled with imidacloprid and other agents.

**VI. Seedling Uprooting and Transplanting**

**Seedling Uprooting**

One day before uprooting the seedlings, water the seedbed thoroughly to make the soil moist and soft, which is convenient for uprooting. When uprooting, try to minimize damage to the root system of the seedlings and maintain the integrity of the root system.

**Transplanting**

Uproot the seedlings and transplant them in a timely manner. During transplanting, ensure shallow and even planting. The planting depth is generally 2 - 3 cm. Determine the planting density reasonably according to factors such as the variety and soil fertility. Generally, plant 20,000 - 30,000 holes per mu for conventional rice and 15,000 - 20,000 holes per mu for hybrid rice.

**4. Y4 Machine - transplanted Rice Seedling Cultivation with Micro - sprinkler Irrigation on Hard Substrate: This is a modern rice seedling cultivation technique, boasting advantages such as land - saving, water - saving, and labor - saving. The following are its key technical points.**

**I. Preparation of the Seedling - raising Site**

**Site Selection**

Choose a hard - surface site with a flat, open terrain, good ventilation, sufficient sunlight, and close proximity to water and power sources, such as a cement floor or a hardened drying yard. This facilitates the installation of micro - sprinkler irrigation equipment and management operations.

**Setting up the Seedling Beds**

Build seedling beds on the hard ground using bricks or other means. The width of the bed is generally 1.5 - 1.8 meters, and the length is determined according to the site conditions. Leave a 30 - 40 - centimeter aisle between beds to facilitate personnel operation and drainage. Set up retaining ridges around the seedling beds, with a height of 10 - 15 centimeters, to prevent the loss of substrate and water.

**II. Substrate Preparation**

**Substrate Selection**

Select a specialized rice seedling - raising substrate with loose texture, good air permeability, strong water - and fertilizer - retention capabilities, and rich in organic matter and various nutrients. Alternatively, the substrate can be prepared independently. Generally, peat soil, vermiculite, and perlite are mixed in a volume ratio of 3:1:1, and an appropriate amount of organic fertilizer and compound fertilizer are added.

**Substrate Treatment**

Sieve the selected substrate to remove impurities and large particles. Then, disinfect it using a fungicide such as carbendazim. Use 100 - 150 grams of 50% carbendazim wettable powder per cubic meter of substrate, dilute it with water, spray it evenly on the substrate, mix well, and stack it for 24 hours before use.

**III. Installation of the Micro - sprinkler Irrigation System**

**Sprinkler Head Arrangement**

Based on the size and shape of the seedling beds, arrange the micro - sprinkler heads reasonably. Generally, the distance between sprinkler heads is 1 - 1.5 meters, and the row spacing is1.2 - 1.5 meters to ensure uniform water spraying without dead corners. The installation height of the sprinkler heads is preferably 30 - 50 centimeters from the surface of the seedling bed, and it can be adjusted according to the actual situation.

**Pipeline Laying**

The main pipeline should be laid along the edge of the seedling - raising site. The branch pipelines are perpendicularly connected to the main pipeline and lead to each seedling bed. The pipelines should be installed firmly to avoid water leakage and blockage. Install filters, regulating valves, pressure gauges, and other equipment on the main pipeline to ensure the normal operation of the micro - sprinkler irrigation system.

**IV. Sowing**

**Seed Treatment**

Similar to the seed treatment method in wet - nursery rice seedling cultivation, it includes steps such as seed - drying, seed - selection, seed - soaking and disinfection, and germination - promotion to bring the seeds to a good germination state.

**Sowing Rate**

Determine the sowing rate according to the variety characteristics and seedling - raising requirements. Generally, 150 - 200 grams of conventional rice seeds are sown per square meter, and 100 - 120 grams of hybrid rice seeds are sown per square meter.

**Sowing Operation**

Evenly fill the seedling beds with the treated substrate to a thickness of 2 - 3 centimeters. Then, evenly broadcast the germinated seeds on the substrate, cover them with another layer of 0.5 - 1 - centimeter - thick substrate, and gently press to ensure full contact between the seeds and the substrate.

**V. Seedling Management**

**Water Management**

Immediately start the micro - sprinkler irrigation system to spray water after sowing to fully moisten the substrate, which is beneficial for seed germination. During the germination period, keep the substrate moist but not waterlogged. Generally, spray water 2 - 3 times a day, and each time the amount of water sprayed should be sufficient to soak the substrate without flowing out of the seedling bed. During the growth of the seedlings, adjust the amount and frequency of water spraying according to the weather and substrate humidity. Increase the frequency of water spraying on sunny and hot days, and reduce it on cloudy or cold days.

**Temperature Management**

Pay attention to weather changes. In case of low temperatures, take heat - preservation measures such as covering with plastic film. In case of high temperatures, cool down by increasing the frequency of water spraying and ventilation. Generally, it is advisable to maintain the temperature of the seedling - raising environment at 20 - 30°C.

**Fertilization Management**

At the 1 - leaf - 1 - heart stage and 3 - leaf - 1 - heart stage of the seedlings, carry out top - dressing in combination with water spraying. Water - soluble compound fertilizers can be selected. Dissolve the fertilizers and prepare a 0.2% - 0.3% solution for spraying. Spray 100 - 150 grams of the solution per square meter each time to meet the nutrient requirements of the seedling growth.

**Pest and Disease Control**

Regularly check the growth of the seedlings and promptly prevent and control pests and diseases. Since the seedling - raising environment on the hard substrate is relatively clean, the occurrence of pests and diseases is relatively low. However, still pay attention to preventing and controlling common pests and diseases such as damping - off, bacterial wilt, and rice thrips. Appropriate pesticides can be used for spraying control.

**VI. Seedling Uprooting and Transplanting**

**Seedling Uprooting**

Stop spraying water one day before uprooting the seedlings to make the substrate appropriately dry, which is convenient for uprooting. When uprooting, use a spade or a special seedling - uprooting tool to start from one end of the seedling bed and shovel up the seedlings together with the substrate, trying to keep the substrate intact and the root system undamaged.

**Transplanting**

Transplant the uprooted seedlings to the main field in a timely manner. During transplantation, handle the seedlings with care, plant them according to the predetermined plant and row spacing, and ensure that the planting depth of the seedlings is appropriate, generally about 2 - 3 centimeters for the roots to be buried in the soil.
